# Sterile inflammation in laminopathies

**DOI:** 10.1016/j.ejcb.2025.151512

**Published:** 2025-08-29

**Authors:** Rafael Cancado de Faria, Susana Gonzalo

**Affiliations:** Edward A. Doisy Department of Biochemistry and Molecular Biology, St Louis University School of Medicine, St Louis, MO 63104, United States

**Keywords:** Sterile inflammation, Laminopathy, HGPS, Cytosolic DNA, DNA sensing, Genome instability, CGAS-STING

## Abstract

Sterile inflammation, an immune response triggered in the absence of pathogens, plays a key role in various chronic diseases, including aging-related disorders, cancer, and autoimmune conditions. This process is driven by damage-associated molecular patterns, such as self-DNA in the cytosol, which activate innate immune pathways and contribute to persistent inflammation. Chronic activation of these pathways exacerbates tissue damage and accelerates disease progression. Recent studies have connected sterile inflammation to laminopathies, a group of genetic disorders caused by mutations in the *LMNA* gene, which encodes nuclear intermediate filament proteins essential for nuclear structure and function. In this review we discuss the molecular mechanisms underlying sterile inflammation in laminopathies, emphasizing self-DNA sensing, inflammatory signaling cascade activation, and their pathological consequences. Additionally, we explore potential therapeutic strategies aimed at modulating inflammation and improving disease outcomes. Understanding these interactions may provide new avenues for targeting inflammation in laminopathies and related conditions.

## 1. Introduction

**Fig. 1.**
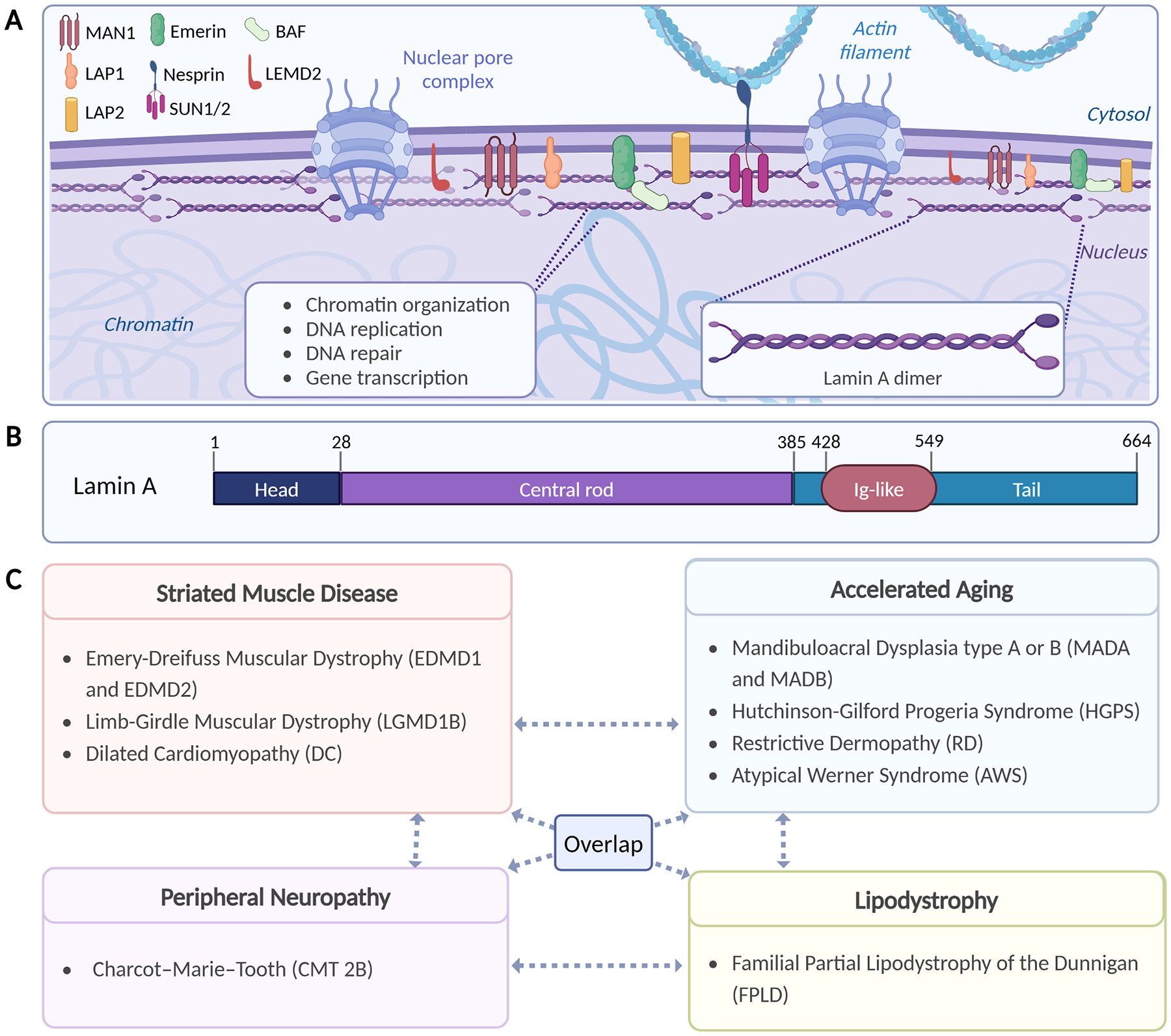
Structure, function, and pathological implications of Lamin A mutants. (A) Schematic representation of Lamin A at the nuclear lamina: Lamin A is localized beneath the inner nuclear membrane, existing in monomeric, dimeric, or polymeric forms. It serves as a scaffold for the binding of various nuclear membrane and soluble proteins, including the LINC complex formed by Nesprins and SUN1/2, as well as BAF, Emerin, LEMD2, LAP1, LAP2, MAN1, among others. Through its interactions with nuclear proteins and chromatin, Lamin A regulates key nuclear processes, such as chromatin organization, gene transcription, DNA replication, and DNA repair. (B) Lamin A protein consists of distinct structural domains: the head, central rod, immunoglobulin (Ig)-like, and tail domains. Mutations in the *LMNA* gene frequently affect the C-terminal regions, including the Ig-like domain, which is critical for interactions with multiple nuclear proteins. (C) Laminopathies: mutations in *LMNA* give rise to a broad spectrum of diseases, generally classified into four overlapping categories: (i) striated muscle disorders, (ii) premature aging syndromes, (iii) peripheral neuropathies, and (iv) lipodystrophies. Despite this classification, substantial phenotypic overlap is frequently observed among these disease groups.

Laminopathies are a group of diseases caused by mutations or altered expression of genes encoding nuclear envelope proteins, particularly lamins and their associated proteins. Lamins are intermediate filament proteins that assemble into a mesh-like structure beneath the inner nuclear membrane, forming the nuclear lamina (Fig. 1A) (Shin and Worman, 2022a). A whole plethora of lamin-interacting proteins have been identified, which cooperate with lamins in tethering chromatin to the nuclear periphery and regulating nuclear processes such as transcription, DNA replication, and DNA repair. These include lamina-associated polypeptides LAP1 and LAP2, transmembrane proteins Emerin and MAN1, and many other nuclear envelope transmembrane proteins known as NETs. Lamins also have an active role communicating the nucleoskeleton with the cytoskeleton via the LINC protein complex; composed of SUN-domain proteins at the inner nuclear membrane (INM) and KASH-domain proteins (Nesprins) at the outer nuclear membrane (ONM). SUN and Nesprins proteins physically interact in the lumen of the nuclear envelope (NE), establishing a direct mechanical link between the nucleus (nuclear lamina) and the cytoplasm (cytoskeleton) (Gonzalo et al., 2017). Two different types of lamins’ networks form at the nuclear lamina: one with A-Type lamins, primarily lamin A and lamin C, encoded by *LMNA* gene; and another with B-type lamins, lamin B1 and lamin B2, encoded by *LMNB1* and *LMNB2* genes, respectively. Structurally, lamins proteins have a central α-helical rod domain that mediates filament formation, which is flanked by non-helical N-terminal and C-terminal domains (head and tail domains), involved in protein/DNA interactions (Fig. 1B). According to the public LOVD database (https://www.lovd.nl/LMNA), over 500 mutations in the *LMNA* gene have been associated with over twenty different diseases, ranging from muscular dystrophies and lipodystrophies, to accelerated aging diseases (Fig. 1C). A specially devastating laminopathy, Hutchinson Gilford Progeria Syndrome (HGPS), has received much attention as a model to unveil molecular mechanisms of aging. While few mutations in *LMNB* genes have been linked to diseases, changes in the lamin B1/B2 protein levels have a significant impact in diseases such as cancer and neurodegeneration (Ahn et al., 2019; Dobrzynska et al., 2016; Shin and Worman, 2022b).

In the last 20 years, we have learned a great deal about lamins functions, not only as architectural nuclear proteins that maintain nuclear plasticity and integrity, but also as participants in many nuclear events. Lamins contribute to the compartmentalization of the genome in the 3D nuclear space, while maintaining genome stability via their participation in DNA repair, DNA replication, and telomere maintenance. Lamins also modulate chromatin structure and epigenetic mechanisms, in addition to being key factors in mechanotransduction. Moreover, much of the impact of lamins alterations in disease comes from changes in gene expression. Either directly or indirectly, lamins play a major role regulating the transcriptome (Gonzalez-Suarez et al., 2009; Graziano et al., 2021, 2018). The consequences of lamins dysfunction at a cellular, tissue, and organismal level have been thoroughly discussed in many excellent reviews. Here, we are focusing on new mechanisms that are activated in laminopathies, and the lines of evidence to suggest that they contribute to disease pathologies. We will review new studies on the role that sterile inflammation, activated by the buildup of self-DNA at the cytoplasm, plays in laminopathies.

## 2. Mechanisms of sterile inflammation

The innate immune system serves as the frontline defense against harmful conditions. Pattern recognition receptors (PRRs) can identify and discriminate “non-self” molecules derived from pathogens, called pathogen-associated molecular patterns (PAMPs). However, multiple PRRs can also initiate an immune response to “self” molecules from the host, named damage-associated molecular patterns (DAMPs) (Chen and Nuñez, 2010). Damaged or dying cells are the producers of an increasing number of DAMPs, including nucleic acids, proteins, ions, glycans, and metabolites. DAMPs trigger inflammation through classical PRRs, including Toll-like receptors (TLRs), NOD-like receptors (NLRs), retinoic acid-inducible gene 1 (RIG-I)-like receptors (RLRs), C-type lectin receptors (CLRs), and intracellular DNA sensors. As DAMPs-driven inflammatory response takes place without pathogens, it is referred to as sterile inflammation (Rock et al., 2010). Like inflammation caused by pathogens, DAMPs can activate PRRs in immune cells and non-immune cells such as epithelial, endothelial, and fibroblasts. Following DAMPs recognition, PRRs activate downstream signaling, including mitogen-activated protein kinase (MAPK), nuclear factor-κB (NFκB), and type I interferon (IFN) pathways, resulting in the production of multiple pro-inflammatory cytokines and chemokines. These trigger the recruitment of inflammatory cells and the activation of an adaptive immune response (Gong et al., 2020).

Activation of sterile inflammation is a common response to alterations in the nuclear architecture, as in the loss of lamin A/C expression (En et al., 2024; Mehl et al., 2022) or mutations in the *LMNA* gene (Bernasconi, 2015; Brayson et al., 2019; Cappelletti et al., 2020; Gerbino et al., 2021; Kreienkamp et al., 2018; Padhiar et al., 2024). Patients carrying pathogenic *LMNA* mutations segregating with cardiomyopathies show elevated plasma levels of interleukins, including IL-1β, IL-6 and IL-8, and circulating granulocyte colony-stimulating factor (G-CSF) (Gerbino et al., 2021). In addition, pro-inflammatory cytokines are differentially expressed in muscular dystrophies linked to mutations in *LMNA* gene and those caused by other mutations (Bernasconi, 2015). In HGPS, macrophages are present at the arterial lesions of progeria patients, and inflammatory markers are present in cells and tissues of animal models (Olive et al., 2010). For instance, genes encoding inflammatory cytokines are upregulated in the liver of progeria mice, including *Il6, Cxcl1, Cxcl2, Ccl8, and Tnf*. Elevated levels of inflammatory cytokines are also observed in mouse serum (IL-6, CXCL1, and TNF-α) (Osorio et al., 2012) and in cells from different tissues, including pre-adipocytes (Hartinger et al., 2023), vascular smooth muscle cells (VSMCs) (Coll-Bonfill et al., 2023), endothelial cells (Atchison et al., 2020), and skin fibroblasts (Kreienkamp et al., 2018; Liu and Henriques, 2019). Moreover, transcriptomic analysis of fibroblasts from HGPS patients compared to normal fibroblasts from their parents revealed an upregulation of nearly 50 genes associated with an antiviral/innate immune/IFN-like response. These include genes encoding for PRRs (*RIG-I*, *MDA5*, *OASs*, and *TLR3*), PRR effectors (*MYD88*, *IRF1/7/9*, *STAT1*, and *NFκB*), and STAT1-regulated IFN-stimulated genes (ISGs) such as *ISG15, MX1, APOBECs, IFIT3*, and *SAMHD1*, among others (Kreienkamp et al., 2018). The current view is that all these inflammatory cascades contribute to disease progression in HGPS and other laminopathies. However, we lack strong direct evidence to support this idea and have also limited knowledge about the molecular mechanisms involved. We discuss below emerging studies that address these gaps of knowledge and use the new findings to target sterile inflammation as a therapeutic strategy in laminopathies.

## 3. Self-DNA sensing: the trigger of sterile inflammation

A key event in sterile inflammation is the aberrant recognition of self-DNA as foreign. Foreign DNA can be recognized by distinct DNA sensors localized in different intracellular compartments, triggering a fine-tuned innate and subsequent, adaptive immune response to eliminate the pathogen (Mathis, 2024; Schlee and Hartmann, 2016). DNA recognition is sequence-independent for most sensors, which enhances the detection of a wide range of pathogens. Likewise, DNA sensors can initiate an aberrant inflammatory cascade in response to self-DNA generated under stress conditions, fueling chronic maladies such as cardiovascular and metabolic diseases, neurodegeneration, autoimmune disorders, and cancer (Dong and Fitzgerald, 2024). These diseases are normally associated with aging, and as such, the term inflammaging has been used to define a condition of elevated blood inflammatory markers that is a risk factor for chronic morbidity, disability, frailty, and premature death. Inflammaging is considered by many as one of the pillars of the biology of aging (Hodes et al., 2016).

**Fig. 2.**
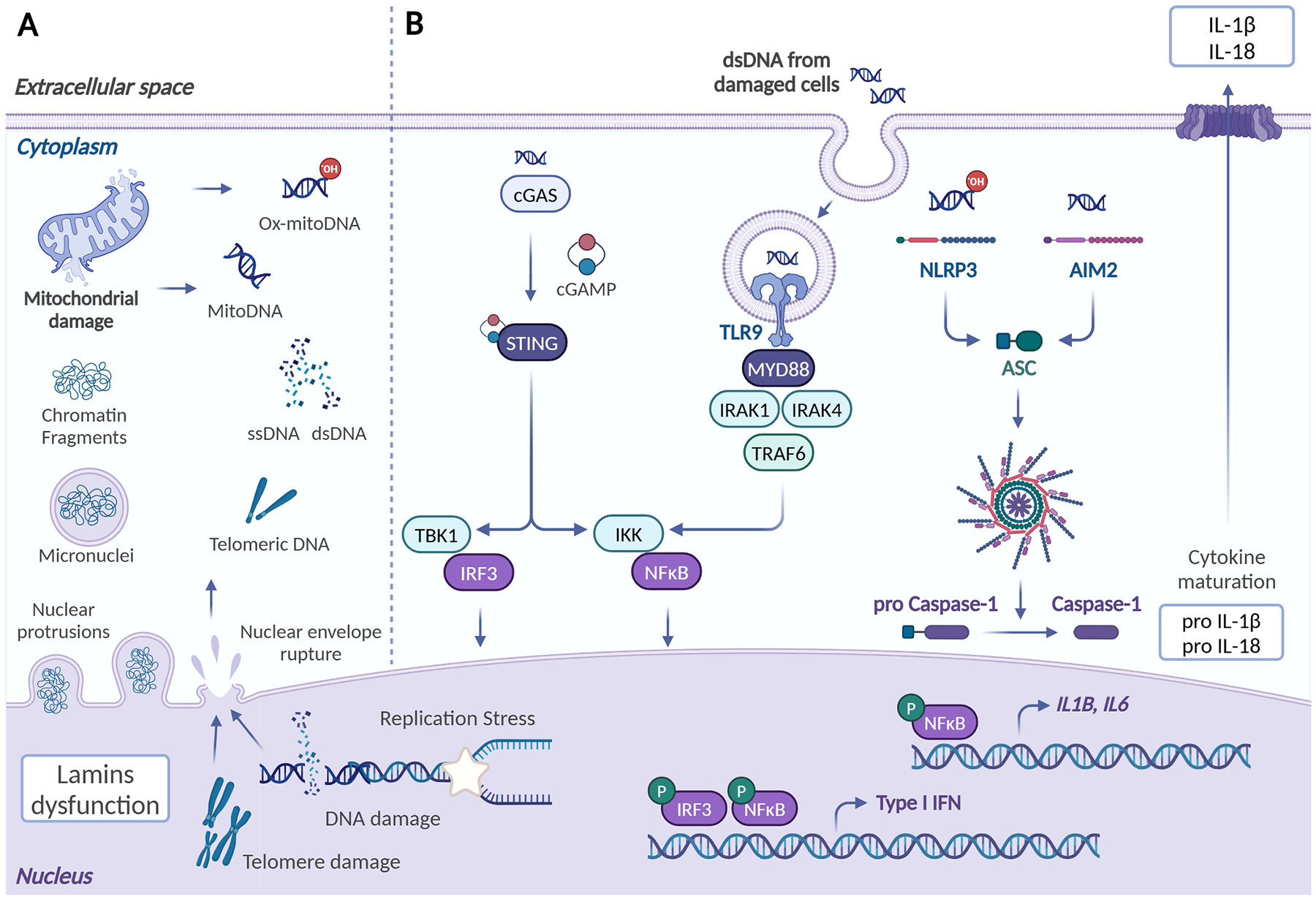
Maladaptive activation of cytosolic DNA sensing pathways by Lamin A dysfunction. (A) Genomic instability and cytoplasmic DNA accumulation: Mutations or loss of Lamin A induce genomic instability, characterized by increased DNA breaks and replication stress, either throughout the genome or specifically at telomeric regions. This DNA damage, in combination with nuclear fragility and impaired cell division, promotes the formation of nuclear protrusions, nuclear rupture, and buildup of self-DNA in the cytoplasm. Cytoplasmic DNA includes micronuclei, nuclear ssDNA or dsDNA, chromatin fragments, and telomeric DNA. Concurrently, mitochondrial dysfunction, frequently observed in laminopathies, also leads to accumulation of mtDNA in the cytosol. These cytosolic DNA species act as DAMPs, which are recognized by PRRs, triggering inflammatory responses. (B) Cytosolic DNA sensors and inflammatory signaling pathways in laminopathies: (i) cGAS: cytosolic dsDNA is recognized by cGAS, which canonically activates STING via cGAMP production. STING, in turn, recruits and activates either TBK1 or IKK, leading to activation of the transcription factors IRF3 and NFκB, which translocate to the nucleus for transcription of pro-inflammatory cytokines. (ii) TLR9: extracellular or engulfed self-DNA from neighboring damaged cells is detected by TLR9, which recruits the adaptor protein MYD88. This leads to the assembly of a complex IRAK1/4 and the E3 ubiquitin ligase TRAF6, activating the IKK-NFκB signaling cascade and inducing the expression of inflammatory mediators. (iii) AIM2- and NLRP3-inflammasome: AIM2 sensor detects cytosolic dsDNA, while NLRP3 senses oxidized mitochondrial DNA (Ox-mtDNA). Upon DNA recognition, both sensors recruit the ASC, promoting oligomerization and the assembly of the inflammasome complex. The inflammasome activates caspase-1, which processes and matures the pro-inflammatory cytokines IL-1β and IL-18 prior to their secretion.

Over the years, multiple cytosolic DNA species have been reported to drive sterile inflammation, including micronuclei, chromatin fragments, mitochondrial DNA (mtDNA), DNA-RNA hybrids or R-loops, single-stranded DNA, and retrotransposons (Chatzidoukaki et al., 2021; Miller et al., 2021; Weinreb et al., 2021). In laminopathies, alterations in the nuclear envelope are often accompanied by chromatin protrusions or nuclear blebbing, leakage of chromatin fragments to the cytosol, and presence of micronuclei (Fig. 2A). In HGPS, for example, telomeric fragments, chromatin, and micronuclei accumulate in the cytosol due to nuclear fragility, impaired mitosis, and defects in DNA replication and repair. Mitochondrial dysfunction and defective mitophagy also contribute to the buildup of mtDNA in the cytoplasm, further increasing the burden of cytosolic self-DNA in HGPS (Cheedipudi et al., 2022; Earle et al., 2020; Kreienkamp et al., 2018; Li et al., 2024; Mu et al., 2020).

For the most part, which DNA sensing pathway is induced by the different cytosolic DNA species remains poorly understood (MacDonald et al., 2023; Takaki et al., 2024), as well as the mechanisms whereby each DNA sensor regulates inflammatory signaling (Dong and Fitzgerald, 2024). In laminopathies, different classes of DNA sensors participate in inflammatory signaling (Fig. 2B). Among them, AIM2 and NLRP3 sensors, activators of inflammasome signaling, and cGAS, activator of STING, are the best characterized. Others such as DHX and DDX helicases, DNA-PK, TLRs, and IFI16 can also sense self-DNA and activate inflammatory programs in laminopathies.

### 3.1. AIM2 and NLRP3 sensors and inflammasome activation

One of the most conserved molecules in living organisms, AIM2, recognizes the phosphodiester backbone of dsDNA in a sequence-independent manner and assembles into an enzymatically active complex termed “inflammasome” (Fig. 2B). AIM2 protein is structurally composed of a C-terminal hematopoietic interferon-inducible nuclear (HIN)-200 domain, and a N-terminal pyrin domain (PYD) (Dong and Fitzgerald, 2024). The HIN-200 domain binds to and oligomerizes along dsDNA while PYD domain recruits the adapter ASC via PYD:PYD interactions. Self-assembled inflammasome, via ASC, recruits and activates caspase 1, leading to proteolytic maturation of proinflammatory cytokines IL-18 and IL-1β and often to cell death via pyroptosis (Lugrin and Martinon, 2018; Sharma and de Alba, 2023). Although AIM2 inflammasome activation has been associated with atherosclerosis (Paulin et al., 2018; Soehnlein and Tall, 2022), chronic heart failures (Ruppert et al., 2020), and diabetic cardiomyopathy (Wang et al., 2019), less is known about its role in laminopathies. AIM2 is considered a key DNA sensor driving inflammasome activation when there is disruption of the nuclear envelope (Fig. 2B) (Di Micco et al., 2016). For instance, pharmacological blockage of lamin A maturation hinders nuclear envelope integrity and facilitates the release of nuclear DNA to the cytosol. In this context, depletion of AIM2 impaired inflammasome activation and secretion of IL-1β, suggesting that AIM2 is a guardian of nuclear integrity and a potential target to reduce sterile inflammation in laminopathies.

Similarly to AIM2, NOD-like receptor-pyrin-containing proteins (NLRPs) can assemble into a multiprotein complex and recruit ASC, which activates caspase 1 for proteolytic maturation of cytokines and for pyroptosis (Zheng et al., 2020). NLRP3 is a PRR that activates the inflammasome pathway in response to various physical and chemical stimuli, such as ion fluxes, uric acids, extracellular ATP, protein aggregates, reactive oxygen species (ROS), and damaged mitochondria (Yang et al., 2019). Although NLRP3 can recognize cytosolic DNA, unlike AIM2, NLRP3 triggers the inflammasome by specifically sensing and binding to oxidized mtDNA (Cabral et al., 2023; Xian et al., 2022; Zhong et al., 2018). The release of ox-mtDNA is a consequence of mitochondrial damage and dysfunction, which are persistent problems in different types of laminopathies, as well as during normal aging (Fig. 2B). As such, studies have reported the detrimental effects of NLRP3 inflammasome in cardiomyopathies and premature aging (HGPS), highlighting the clinical relevance of NLRP3 targeting in lamins-associated diseases (González-Dominguez et al., 2021; Muela-Zarzuela et al., 2024; Sun et al., 2022; Zeng et al., 2020). Similarly, emerging evidence suggests that pyroptosis, a highly inflammatory form of programmed cell death activated by inflammasome pathways, contribute to accumulated tissue damage during aging. Consequently, dead cells release different stimuli that amplify inflammation within the tissue environment, enhancing tissue dysfunction (Li et al., 2023). By linking inflammation and cell death, pyroptosis may serve as a key mediator of the chronic inflammatory milieu that underlies aging and its associated disorders (Huang et al., 2022; Liu et al., 2024).

### 3.2. Helicases, DNA-PK, TLR9, and IFI16 sensors of self-DNA

Other DNA sensors described over the years include DHX and DDX helicases, DNA-PK, and IFI16. DNA-PK is a potent DNA sensor that activates the expression of inflammatory cytokines and IFNs (Ferguson et al., 2012). In human cells stimulated with viruses, but not upon DNA damage, DNA-PK can sense cytosolic DNA and trigger a robust antiviral response independent of STING (Burleigh et al., 2020). Likewise, DNA helicases members of DExD/H-Box family can sense DNA and activate the production of type I IFN. Two major subgroups are present in this family: DDX helicases, such as DDX41, which senses transfected dsDNA, cyclic di-GMP, and cyclic di-AMP; and DHX helicases, such as DHX9 and DHX36, which sense CpG-B and CpG-A DNA, respectively (Dong and Fitzgerald, 2024). Another important DNA sensor, IFI16 (Unterholzner et al., 2010), can be found in the nucleus and cytoplasm and trigger both, the IFN response and the ASC- and CASP1-containing inflammasome. In addition, upon nuclear DNA damage, IFI16 can trigger the expression of inflammatory cytokines via STING but independent of cGAMP (Dunphy et al., 2018). Although these DNA sensors are implicated in antiviral responses as well as self-DNA recognition, little is known about their role in laminopathies. For instance, DNA-PK has increased activity in myotubes of *Lmna* knockout mice along with increased DNA damage markers (Earle et al., 2020). However, whether DNA-PK initiates sterile inflammation in lamin A-deficient mice remains to be determined.

Additional sensors of self-DNA include TLRs. In patients with *LMNA*-related myopathies, inflammation has been associated with elevated TLR9 levels in macrophages and skeletal muscle fibers, but not in muscle biopsies from *LMNA*-independent muscular dystrophies such as facioscapulohumeral muscular dystrophy (FSHD) (Cappelletti et al., 2018). This suggests that disruptions in nuclear lamina architecture may have a more determinant role in triggering a TLR9-dependent immune response (Fig. 2B) compared to other neuromuscular genetic disorders. Other studies reported detrimental roles of TLR9 downstream factors in laminopathies (MacRae et al., 2023; Sengupta and Sengupta, 2024). However, for the most part, our knowledge of how these sensors of self-DNA contribute to the pathogenesis of laminopathies is in its infancy.

### 3.3. cGAS sensor of dsDNA

One of the major pathways that mediates the innate immune response to DNA is regulated by cGAS (Fig. 2B). Localized at both the cytosol and the nucleus, cGAS is a dsDNA-sensing enzyme that identifies DNA by its phosphodiester backbone regardless of sequence specificity. Cytosolic DNA binds and activates cGAS enzymatic activity, catalyzing the synthesis of 2′3′-cyclic GMP–AMP (2′3′-cGAMP) using adenosine 5′-triphosphate (ATP) and guanosine 5′-triphosphate (GTP) as substrates. cGAMP, a second messenger, rapidly diffuses throughout the cell and binds to the adaptor protein STING at the endoplasmic reticulum (ER) membrane. Under normal conditions, once bound to cGAMP, STING undergoes conformational change, oligomerization, a variety of post-translational modifications, and translocation to Golgi, ERGIC, and other membranous organelles (broadly named perinuclear compartment or PNC). At the PNC, STING forms a ternary complex with the kinase TBK1 and the transcription factor IRF3. STING also interacts with IKK to activate the transcription factor NFκB. Phosphorylation of IRF3 and NFκB allows for their translocation to the nucleus and expression of inflammatory cytokines and type I IFN (Dong and Fitzgerald, 2024; Dvorkin et al., 2024). This is considered the canonical cGAS-STING pathway of activation of sterile inflammation. Interestingly, STING is also activated by cGAMP via intracellular or intercellular signaling, suggesting that cGAMP acts as “immunotransmitter”, capable of diffusing through gap junctions, and engaging and activating STING in neighbor cells (Chen et al., 2016; Ritchie et al., 2022). The cGAS-STING signaling cascade is a potent antiviral pathway that is tightly regulated by multiple mechanisms, including cGAMP degradation, cGAS cleavage, STING degradation, and inhibitory cGAS and STING post-translational modifications. However, its persistent activation is associated with multiple chronic diseases, such as diabetes, cardiovascular disease, autoimmune diseases, neurodegeneration, cancer, as well as normal and premature aging (Geng et al., 2023; Gulen et al., 2023; Kreienkamp et al., 2018; Luo et al., 2023; Samson and Ablasser, 2022; Xiong et al., 2023).

Despite growing evidence linking cytosolic DNA sensing to inflammation in laminopathies, our understanding of these mechanisms remains limited. Different *LMNA* mutations may lead to distinct forms of genomic instability, mitochondrial dysfunction, or even increased retrotransposon expression, potentially activating different innate immune sensors. Furthermore, the same *LMNA* mutation could elicit divergent effects across various cell types, such as adipocytes, myofibroblasts, cardiomyocytes, and immune cells. Therefore, to unravel these complexities, future research should focus on studying the activation of different cytosolic DNA sensors and their downstream signaling cascades in each specific context and cell type, ideally using isogenic cellular models. Critically, further mechanistic studies are needed to elucidate the downstream consequences of sensing self-DNA in the cytoplasm, shedding light on its role in disease progression and identifying potential therapeutic targets in laminopathies.

## 4. Role of cGAS-STING pathway driving inflammation in laminopathies

**Fig. 3.**
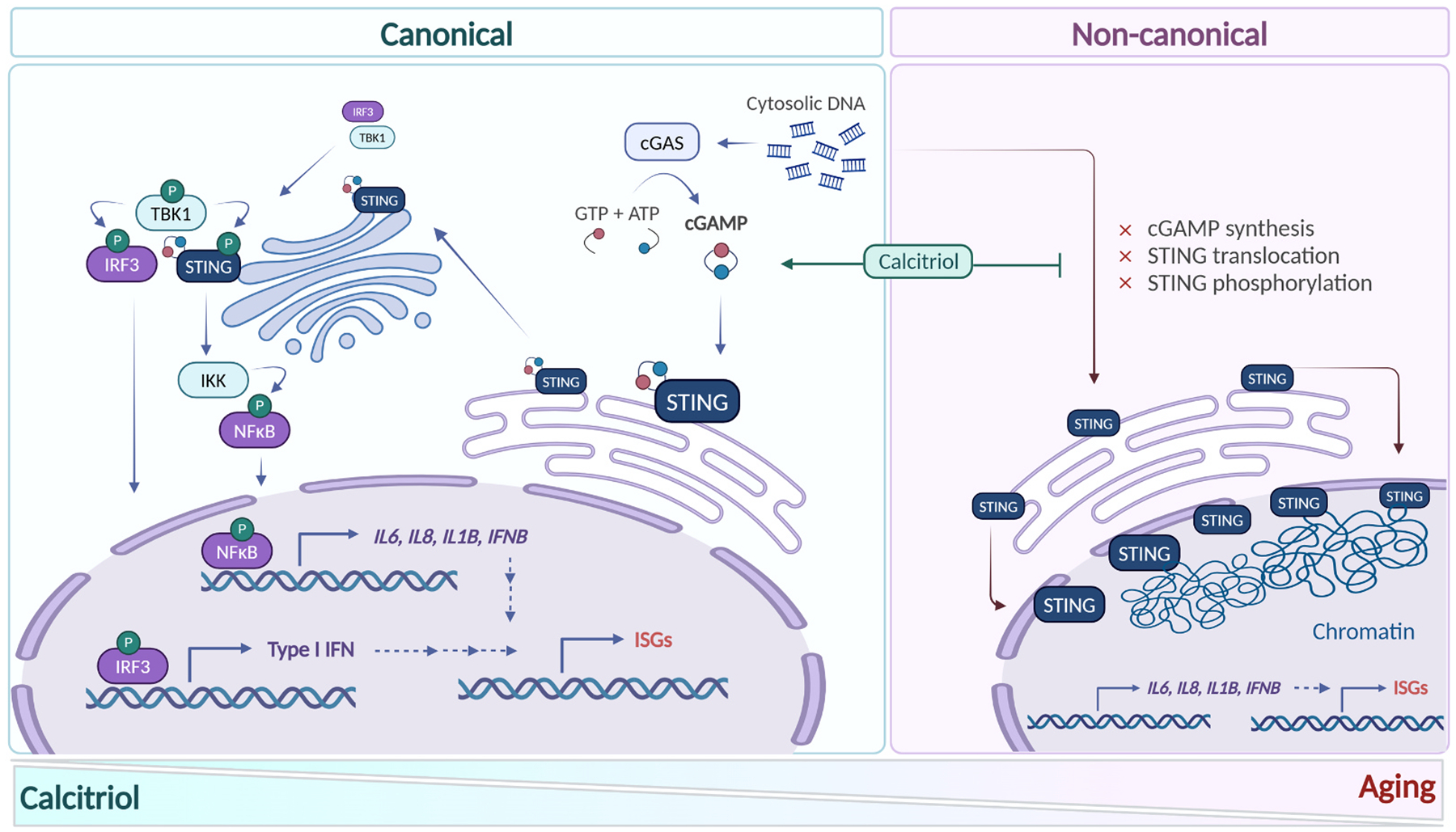
Differential activation of the cGAS-STING pathway during progeroid laminopathy and aging. In a normal young cell, the accumulation of cytosolic DNA activates the canonical cGAS-STING pathway. This canonical activation involves the synthesis of cGAMP by cGAS, which subsequently binds to STING. Upon activation, STING translocates to the Golgi apparatus, where it forms a complex with TBK1-IRF3 or IKK-NFκB. These complexes undergo phosphorylation, leading to the translocation of IRF3 and NFκB to the nucleus, where they drive the transcription of pro-inflammatory molecules. During aging and laminopathic aging (HGPS), cells progressively lose their capacity to activate the canonical cGAS-STING pathway, despite the accumulation of cytosolic DNA. In aging and HGPS, cGAS and STING continue to promote sterile inflammation but via a non-canonical cGAS-STING pathway. This alternative signaling mode does not rely on robust cGAMP synthesis, STING phosphorylation, or its trafficking to the Golgi. In contrast, STING remains predominantly associated with the endoplasmic reticulum and the nuclear envelope, while also becoming enriched at chromatin sites in aged and progeroid cells. Notably, activation of vitamin D receptor (VDR) signaling by calcitriol treatment has been shown to enhance the ability of aged and progeroid cells to activate the canonical cGAS-STING pathway in response to synthetic DNA. Concurrently, VDR signaling suppresses inflammation driven by the non-canonical cGAS-STING pathway, thereby mitigating chronic inflammatory responses associated with cellular aging.

Our studies in HGPS have shown that the combination of nuclear fragility and genomic instability due to replication stress, lead to the buildup of self-DNA in the cytoplasm (Fig. 3). This is accompanied by increased expression of cGAS and STING, and a robust sterile inflammation response. These features seem to play a role in the disease because a variety of pharmaceutical compounds used in preclinical studies for HGPS therapy not only alleviated disease phenotypes but also decreased DNA replication problems and sterile inflammation (Kreienkamp et al., 2018). Importantly, our most recent data show that STING depletion suppresses cytokines and *IFNB* expression in progeria, suggesting a major involvement of STING in sterile inflammation in this laminopathy. Unexpectedly however, we found that STING-driven inflammation in HGPS occurs via an alternative/non-canonical mechanism (Fig. 3). Despite the abundance of cytosolic DNA, progeria cells do not exhibit classical markers of the canonical cGAS-STING pathway such as elevated cGAMP synthesis, STING trafficking to PNC, and phosphorylation of STING, TBK1, and IRF3. Moreover, stimulation of the canonical cGAS-STING pathway using exogenous synthetic dsDNA [poly (dA:dT)] revealed a resistance of progeria cells to activate the canonical cGAS-STING pathway. This was evidenced by a marked reduction in cGAMP production, in STING localization at PNC, and in STING phosphorylation, compared to normal cells. Instead, STING appears mislocalized upon dsDNA stimulation, suggesting that the alternative STING behavior in progeria cells is a potential barrier for canonical cGAS-STING activation. Interestingly, rejuvenation of progeria cells with calcitriol (vitamin D receptor signaling activator) suppresses the non-canonical cGAS-STING pathway while rescuing the canonical pathway in response to synthetic DNA. These findings unveil a novel mechanism regulating the canonical cGAS-STING pathway in progeria. Importantly, deficiencies in the canonical cGAS-STING pathway in progeria cells are reproduced in primary cells during replicative senescence or DNA damage-induced senescence, indicating a conserved mechanism occurring in aging/senescent cells.

Altogether, these findings suggest that during aging, cells are hindered in their ability to activate the canonical cGAS-STING pathway, which is critical for the cells to respond to pathogen invasion. These deficiencies might be caused by alterations in lamins known to occur in aging contexts, such as the progressive loss of lamin B1 expression during senescence, or the altered maturation of lamin A in progeria. Lamins have been shown to interact with STING at the INM, regulating its translocation to the ONM and to the ER (Dixon et al., 2021). It is possible that mutations such as progerin sequester STING at the NE and in nuclear invaginations, reducing the ability of STING to participate in the canonical pathway in response to cytosolic DNA. How the loss of lamin B1 during senescence impacts STING localization and function is not known. In summary, recent studies are opening new horizons to investigate how lamins participate in the altered behavior of STING in aging, and the role/s that the non-canonical cGAS-STING pathway plays in the pathophysiology of laminopathies and other related diseases.

In addition to genomic cytosolic DNA, recent studies have explored the role of mtDNA leakage into the cytoplasm and its contribution to cGAS-STING pathway activation in HGPS. In a study using myofibers from the *Zmpste24*^−/−^ progeria mouse model (with defective maturation of lamin A protein) researchers observed increased mtDNA release from damaged mitochondria, impaired mitophagy, and heightened activation of the cGAS-STING signaling pathway. Voltage-dependent anion channel 1 (VDAC1), the most abundant protein in the mitochondrial outer membrane, was found to undergo oligomerization, increasing mitochondrial membrane permeability and mtDNA release into the cytosol. Pharmacological inhibition of VDAC1 oligomerization effectively reduced mtDNA release, suppressed cGAS-STING activation, and diminished the expression of the senescence-associated secretory phenotype (SASP) (Li et al., 2024). Similarly, another study demonstrated the involvement of the STING-NFκB axis in response to mtDNA leakage in mesenchymal stem cells derived from HGPS patients. The authors showed that mitophagy dysfunction in HGPS facilitated the accumulation and cytosolic release of mtDNA, leading to STING-NFκB activation. Since mitophagy plays a crucial role in mitochondrial quality control by clearing damaged mitochondria, its impairment can contribute to chronic inflammation and cellular dysfunction. Interestingly, inducing mitophagy to enhance mitochondrial clearance significantly reduced STING activation and inflammation, while improving progeria-associated phenotypes in mouse models (Sun et al., 2024). Collectively, these studies support the concept that mitochondrial dysfunction drives cGAS-STING activation, in a similar manner as genomic instability and nuclear fragility.

Unlike HGPS, studies with *LMNA*-associated dilated cardiomyopathy have contrasting results. In one report, deletion of mouse *Lmna* in cardiac myocytes causes dilated cardiomyopathy, premature death, myocardial fibrosis, and apoptosis, similarly to what is observed in patients with *LMNA* gene mutations. The authors showed that abrogation of cGAS not only reduced pro-inflammatory cytokines but also extended survival and attenuated cardiac dysfunction, apoptosis, and fibrosis, suggesting the contribution of cGAS to inflammation and disease phenotypes (Cheedipudi et al., 2022). In contrast, a recent *in vivo* study in *Lmna*-associated dilated cardiomyopathy showed no involvement of cGAS-STING pathway, despite increased pervasive nuclear envelope ruptures in cardiomyocytes with loss of lamin A/C. Deletion of *Mb21d1* (gene encoding cGAS) or *Tmem173* (gene encoding STING) did not rescue cardiomyopathy or inflammation in adult mice, likely due to low levels of cGAS expression in cardiomyocytes. In this context, extracellular matrix signaling was a key mechanism driving inflammation and cardiomyopathy (En et al., 2024). Overall, although different groups have demonstrated a detrimental role of cGAS-STING activation in distinct cardiac problems (Luo et al., 2023; Shamseddine et al., 2023; Yan et al., 2022; Zhou et al., 2024), its contribution to inflammation and disease phenotypes might be defined by how specific alterations in lamins functions impact nuclear architecture in different cell types. Additional mechanistic studies are needed to unveil how lamins can regulate the cGAS-STING pathway in diverse lamin-associated diseases.

## 5. Persistent cytokine storm in laminopathies

Activation of DNA sensing pathways leads to the expression and secretion of multiple inflammatory cytokines, including IL-1β, IL-6 and IFNs, creating an inflammatory environment that affects neighbor cells and boosts immune cell responses (Dong and Fitzgerald, 2024). Ideally, inflammation should recede immediately after elimination of an insult to maintain tissue and organismal homeostasis. However, low-grade persistent cytokine secretion occurs in many chronic inflammatory diseases, as well as laminopathies (Bernasconi, 2015; Cheedipudi et al., 2022; En et al., 2024; Kreienkamp et al., 2018; Padhiar et al., 2024; Shen et al., 2013).

### 5.1. Interleukin 1 beta

IL-1β is part of IL-1 family and a significant mediator of inflammation and tissue damage. It is initially translated as pro-IL-1β and processed via caspase-1 activity, as in the inflammasome pathway activation. Once released to the extracellular space, IL-1β binds to IL-1R1 or IL-1RII receptors in different cell types (T cells, epithelial cells, fibroblasts, or endothelial cells), contributing to pro-inflammatory processes (Dinarello, 2009). The intracellular signal transduction involves the recruitment of the adapter protein MyD88, which allows for the activation of IRAK1and TRAF6. This results in the activation of other kinases such as p38 MAPK, and the transcription factor NFκB, inducing the transcription of various inflammatory genes (Balahura et al., 2020).

IL-1β is considered a potential biomarker cytokine in patients with muscular laminopathy, whose IL-1β serum levels are significantly higher compared to healthy controls and other non-muscular laminopathies (Cappelletti et al., 2020). Serum levels of IL-1β are also elevated in patients with cardiolaminopathies, carrying different mutations in the *LMNA* gene, as well as in patients with lamin-associated lipodystrophy (Foss-Freitas et al., 2018; Gerbino et al., 2021). Moreover, models of progeroid diseases caused by *LMNA* mutations have increased IL-1β production. Upregulation of IL-1β expression and secretion occurs in inducible mesenchymal stem cells (iMSCs) and skin fibroblasts from patients with homozygous *LMNA* p.R527C, who have a severe form of mandibuloacral dysplasia (MAD), along with multiple progeroid manifestations (Padhiar et al., 2024). Likewise, HGPS cells and distinct tissues from progeria mouse models have increased production of IL-1β (Cancado de Faria et al., 2023; González-Dominguez et al., 2021; Maynard et al., 2022; Muela-Zarzuela et al., 2024). Although anti-inflammatory therapies that reduce IL-1β signaling/production are beneficial for HGPS, the specific contribution of IL-1β pathway to disease phenotypes in laminopathies is unknown. Future studies can fill this gap by specifically targeting IL-1β cascade with FDA-approved antibodies against IL-1β or IL-1RI, which have proven positive impact on autoimmune and cardiovascular diseases (Dinarello et al., 2012).

### 5.2. Interleukin 6

**Fig. 4.**
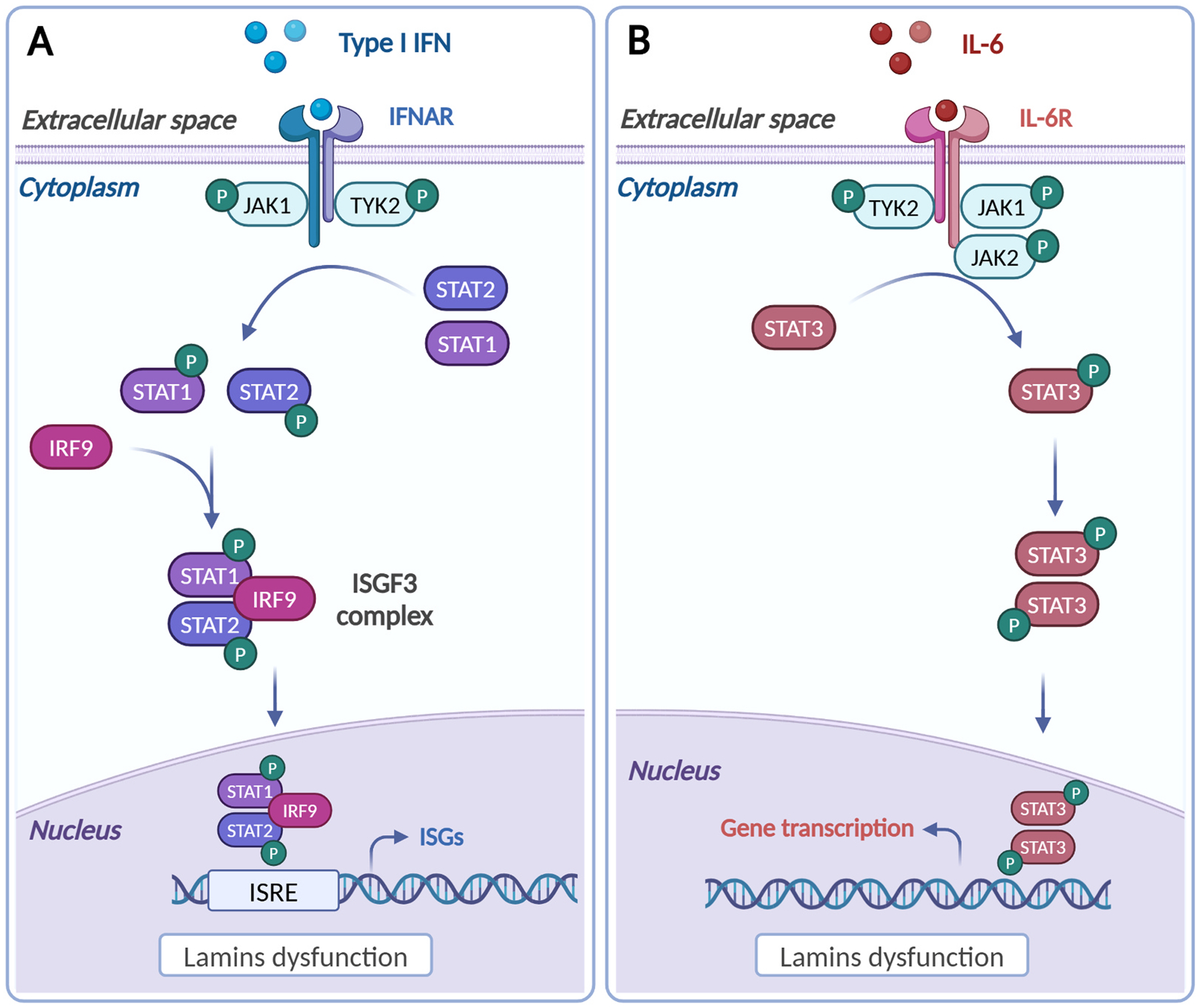
Activation of the JAK-STAT signaling pathway by Lamin dysfunction. Lamin A deficiencies trigger inflammatory cytokine production via aberrant DNA sensing pathways, leading to the release of type I IFNs, such as IFN-β, and IL-6. These cytokines propagate inflammation through autocrine and paracrine signaling, activating the JAK-STAT pathway. (A) Type I IFN response: Type I IFNs bind to their transmembrane receptor, IFNAR, leading to the phosphorylation and autoactivation of JAK1 and TYK2 at the intracellular domain of the receptor. Activated JAK kinases phosphorylate the transcription factors STAT1 and STAT2, facilitating the recruitment of IRF9 to form the interferon-stimulated gene factor 3 (ISGF3) complex. The ISGF3 complex shuttles to the nucleus, where it binds ISREs and induces the transcription of hundreds of ISGs, driving an antiviral and pro-inflammatory cellular state. (B) IL-6-mediated STAT3 activation: IL-6 binding to its receptor (IL-6R) leads to the activation of JAK1, JAK2, and TYK2 through phosphorylation and autoactivation. These kinases subsequently phosphorylate and activate STAT3, promoting its dimerization and nuclear translocation. Once in the nucleus, STAT3 modulates diverse transcriptional programs, amplifying inflammatory responses, altering cellular metabolism, and regulating cell proliferation. Persistent activation of JAK-STAT signaling in laminopathies contributes to chronic inflammation and tissue dysfunction.

Unlike IL-1β, IL-6 and IFN response occur through the JAK kinases and STAT transcription factors (Fig. 4A and B). Canonically, pleiotropic IL-6 binds to its receptor IL-6R, which exists in both soluble and transmembrane forms. IL-6 bound to its receptor subsequently forms a complex with the ubiquitously expressed transmembrane glycoprotein gp130, recruiting and activating JAK1 and JAK2 by cross-phosphorylation. JAK1/2 phosphorylates STAT3, resulting in STAT3 translocation to the nucleus and transcription of many downstream genes whose products participate in development and disease (Fig. 4B) (Kang et al., 2019; Reeh et al., 2019).

IL-6 is upregulated in cells, disease models, and plasma samples of patients with laminopathies, being also suggested as a potential biomarker in Lamin-associated muscular diseases (Cancado de Faria et al., 2023; Cappelletti et al., 2020; Gerbino et al., 2021; Osorio et al., 2012; Padhiar et al., 2024; Squarzoni et al., 2021). Alterations of nuclear architecture in skeletal muscle cells from lamin A/C-deficient mice or mice carrying different *Lmna* mutations cause progressive decline in myofiber health, featured by reduced nuclear stability, chromatin protrusions, and increased IL-6 expression (Earle et al., 2020; Xiong et al., 2020). In addition to muscular dystrophy–like phenotypes, lamin A/C deficiency in skeletal muscles also results in osteoporosis, a critical hallmark in laminopathies and aging. Interestingly, knockout of IL-6 in muscles of lamin A/C-deficient mice diminishes the deficits in trabecular bone mass, suggesting a key role of skeletal muscle lamin A/C and IL-6 in bone loss (Xiong et al., 2020).

In HGPS, blocking elevated IL-6 signaling cascade *in vitro* and *in vivo* significantly improves disease phenotypes (Squarzoni et al., 2021) as discussed below. Specifically, IL-6 has been implicated in the progression of progeria-associated lipodystrophy, and growing evidence suggests its potential involvement in other laminopathic lipodystrophies, including familial partial lipodystrophy of the Dunnigan type (FPLD) (Krüger et al., 2024a). Patients with FPLD exhibit elevated serum levels of IL-6, which has been linked to metabolic syndrome, insulin resistance, and an increased risk of cardiovascular disease, hallmarks of this condition (Foss-Freitas et al., 2018). In a small study involving FPLD patients, gemcabene, a lipid-lowering agent that inhibits IL-1β and IL-6 production, was found to reduce dyslipidemia, suggesting a potential role of IL-6 in this form of lipodystrophy (Akinci et al., 2020; Srivastava et al., 2018). However, further investigation into IL-6 targeting and its systemic effects, as well as its impact on dysfunctional adipocytes in FPLD, is needed to better understand the detrimental mechanisms of IL-6 in laminopathic lipodystrophies.

### 5.3. Interferons

Similar to the IL-6 signaling cascade, the binding of IFNs to their respective receptors activates tyrosine kinases such as JAK1, JAK2, and TYK2 (Fig. 4A). Specifically, type I IFNs—including IFN-α, IFN-β, IFN-δ, IFN-ε, IFN-κ, IFN-τ, and IFN-ω—bind to the transmembrane receptors IFNAR1 and IFNAR2, triggering JAK activation. This leads to the phosphorylation of the transcription factors STAT1 and STAT2, which then form heterodimers and associate with IRF9 to create the IFN-stimulated gene factor 3 (ISGF3) complex. ISGF3 translocates to the nucleus, where it binds to interferon-stimulated response elements (ISREs) in gene promoters, inducing the expression of hundreds of ISGs. Additionally, type I IFNs can activate STAT1 homodimers, a signaling pathway typically associated with type II IFNs, such as IFN-γ, thereby promoting the expression of ISGs regulated by gamma-activated sequence (GAS) elements in gene promoters (McNab et al., 2015; Platanias, 2005).

Over the years, numerous studies have highlighted how immune cells regulate lamin A/C expression and maturation to fulfill their roles in innate and adaptive immunity (Saez et al., 2020). A recent study found that the activation of pro-inflammatory macrophages leads to a loss of lamin A/C at both transcriptional and protein levels, while sustaining elevated pro-inflammatory gene and cytokine expression through the IFN-β-STAT1 signaling pathway. Blocking IFN-β-STAT1 signaling or preventing lamin A/C degradation significantly suppressed the pro-inflammatory macrophage response, underscoring a strong connection between nuclear intermediate filaments and innate immunity in health and disease (Mehl et al., 2022). In contrast to lamin downregulation, neural stem/progenitor cells (NSPCs) exposed to oxidative stress exhibited impaired nuclear lamin maturation, resulting in cytosolic DNA leakage and activation of the cGAS-STING-IFN pathway. Notably, deletion of the C-terminus of mature lamin A diminished the IFN response triggered by oxidative stress, highlighting nuclear lamins as key regulators of oxidative stress responses (Hwang et al., 2021).

In laminopathies, IFNλ2 levels are significantly elevated in the serum of patients with striated muscle laminopathies, including those with Emery–Dreifuss muscular dystrophy 1 (EDMD1), idiopathic dilated cardiomyopathy, and familial dilated cardiomyopathy (Cappelletti et al., 2020). However, the specific role of IFNλ2 in the pathogenesis of *LMNA*-related muscle disorders remains to be explored. In HGPS, severe nuclear architecture alterations are accompanied by increased DNA replication stress, DNA damage, cytosolic DNA buildup, and upregulation of IFN-β and IFN-γ expression (Cancado de Faria et al., 2023; Kreienkamp et al., 2018; Liu and Henriques, 2019). Progerin-expressing cells also show activation of JAK-STAT1 signaling and upregulation of more than 50 ISGs. While type I IFNs and IFNAR have been identified as direct mediators in other progeroid syndromes, their precise role in the IFN response in HGPS remains unclear, often being described as an IFN-like response (Kreienkamp et al., 2018; Lei et al., 2021). Nonetheless, both pharmacological inhibition of the JAK-STAT pathway and genetic depletion of STAT1 have led to significant improvements in HGPS phenotypes, underscoring the crucial role of IFN signaling in laminopathies and aging (Arnold et al., 2021; Cancado de Faria et al., 2023; Griveau et al., 2020; Hartinger et al., 2023; Kreienkamp et al., 2018; Liu and Henriques, 2019).

## 6. Tissue degeneration and inflammation-targeted therapies in HGPS

Inflammation is a double-edged sword for host tissues. While inflammatory responses play a crucial role in protecting against infections and repairing damaged tissues, by preventing pathogen spread and promoting tissue repair, their adequate onset and resolution are essential for tissue maintenance. However, when inflammation becomes prolonged or dysregulated, it can be detrimental, contributing to the development of various chronic diseases (Dong and Fitzgerald, 2024; Ortega-Gómez et al., 2013).

Within laminopathies, HGPS has been strongly associated with persistent tissue inflammation, accelerated tissue degeneration, and overall organismal decline. Studies have reported increased inflammation and fibrosis across multiple organs in progeria mouse models, particularly near the end of their lifespan at approximately 100 days (Coll-Bonfill et al., 2023; Krüger et al., 2024b). Although these mice do not fully recapitulate the severity of human disease phenotypes, they exhibit profound tissue wasting. Total body weight loss is accompanied by significant reductions in muscle mass, adipose tissue, and bone density, along with impairments in energy utilization and expenditure, featuring starvation and cachexia (Kreienkamp et al., 2019). Given that cardiovascular complications are the leading cause of death in HGPS patients, progeria mice also develop histopathological abnormalities in the aorta, including calcification, VSMC loss, medial and adventitial fibrosis, reduced vessel wall thickness, linear and fragmented elastin fibers, and increased inflammation. Moreover, the hearts of progeria mice exhibit pronounced fibrosis in the left ventricle, epicardial, and myocardial regions, further confirming the detrimental cardiovascular effects of progerin (Cancado de Faria et al., 2023; Coll-Bonfill et al., 2023; Hamczyk et al., 2018; Krüger et al., 2024b; Olive et al., 2010). Beyond the cardiovascular system, progerin toxicity also induces skin abnormalities, including excessive fibrotic tissue deposition, reduced dermal thickness, severe loss of subcutaneous fat, and diminished hair follicles, leading to progressive and severe alopecia. Likewise, fibrosis has been observed in the lungs, liver, thymus, kidneys, and intestines of progeria mice, highlighting widespread tissue damage and premature degeneration across multiple organs (Cancado de Faria et al., 2023; Kreienkamp et al., 2019; Krüger et al., 2024b).

Notably, interventions that mitigate tissue wasting have been shown to extend lifespan. A particular study demonstrated that introducing a high-fat diet (HFD, 60 % of calories from fat) to progeria mice nearing the end of their lifespan significantly improved morbidity and delayed early lethality. Mice switched to the HFD exhibited increased body weight, lean mass retention, and preservation of fat tissue, enabling them to survive in good health for an additional 30 days. Remarkably, when progeria mice were placed on HFD immediately after weaning, their lifespan doubled, featuring delayed tissue degeneration and improved overall health (Kreienkamp et al., 2019). These findings suggest that nutritional interventions that support tissue maintenance can play a crucial role in extending health span and longevity in progeria.

**Fig. 5.**
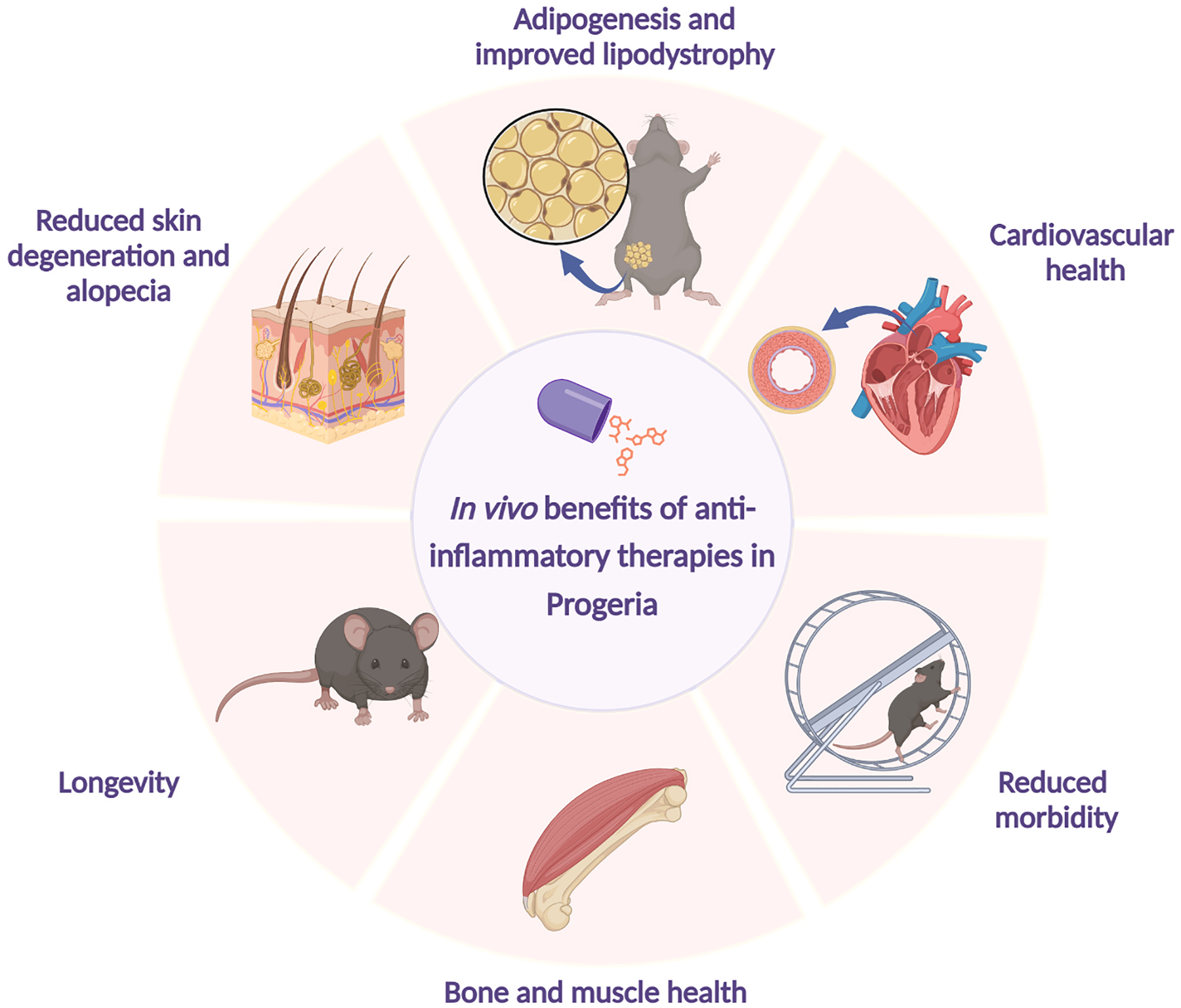
*In vivo* benefits of anti-inflammatory therapies in Progeria. In HGPS, chronic low-grade sterile inflammation disrupts cellular, tissue, and organismal homeostasis, significantly contributing to accelerated aging phenotypes. Targeting sterile inflammation through various therapeutic strategies and inflammatory signaling pathways has demonstrated substantial benefits in preclinical progeria models. Anti-inflammatory interventions have been shown to: (i) enhance adipogenesis and reduce lipodystrophy, thereby improving metabolic function; (ii) decrease morbidity and extend lifespan, promoting overall longevity; (iii) preserve cardiovascular, bone, and muscle health, mitigating the early onset of age-related tissue degeneration; and (iv) delay the progression of alopecia and skin abnormalities, improving skin integrity and appearance. Collectively, these findings underscore the therapeutic potential of mitigating inflammatory pathway toxicity in HGPS, highlighting anti-inflammatory strategies as promising candidates for progeria treatment.

Novel therapeutic approaches, including gene editing, base editing, and antisense oligonucleotides, have successfully extended lifespan, improved health as well as tissue quality in progeria mouse models, representing a promising direction for HGPS treatments (Fong et al., 2009; Koblan et al., 2021a, 2021b; Puttaraju et al., 2021; Santiago--Fernández et al., 2019). Additionally, pharmacological interventions in HGPS, including administration of antioxidants, mTORC1 inhibitors, and the FDA-approved farnesyl transferase inhibitor (Lonafarnib), have demonstrated significant improvements at the cellular, tissue, and systemic levels (Cao et al., 2011; Gabriel et al., 2015; Kubben et al., 2016; Murtada et al., 2023; Suzuki et al., 2023; Vehns et al., 2022; Xiong et al., 2016). Often, these improvements were accompanied by a reduction in inflammatory signatures. In line with these observations, anti-inflammatory therapies such as corticosteroids have gained interest as potential interventions. Corticosteroids are widely used in different inflammatory conditions, such as Duchenne Muscular Dystrophy, where they mitigate inflammation and fibrosis, key drivers of muscle degeneration and functional decline (Al Tanoury et al., 2021; Guglieri et al., 2022; Herbelet et al., 2020). However, despite the potential therapeutic benefit of corticosteroids in laminopathies has been discussed, formal preclinical and clinical studies are necessary to assess their efficacy and long-term impact in these disease contexts. Unlike steroids, preclinical studies have increasingly highlighted the potential of alternative anti-inflammatory interventions in HGPS models. In this section, we discuss key therapeutic targets involved in inflammation and their reported benefits in HGPS (Table 1 and Fig. 5).

### 6.1. Calcitriol treatment

Vitamin D is a vital steroid hormone primarily recognized for its role in maintaining calcium and phosphate homeostasis, which is essential for bone health. Beyond its classical functions, the active form of Vitamin D, 1α,25-dihydroxycholecalciferol (calcitriol), plays a crucial role in inflammatory response regulation, and has been promising for treating chronic diseases, autoimmune disorders, neurodegeneration, and cancer (Colotta et al., 2017; Krishnan and Feldman, 2011). Multiple cell types express the vitamin D receptor (VDR), a nuclear receptor activated by calcitriol binding, that modulates gene expression, including the suppression of pro-inflammatory genes such as *IL-6, IL-8, IFN, IL-1β*, and ISGs, while promoting the expression of anti-inflammatory genes. The downregulation of cytokines and ISGs by calcitriol primarily occurs through the inhibition of key inflammatory pathways such as NFκB, the NLRP3 inflammasome, and JAK-STAT signaling, pathways that have been implicated in the pathogenesis of HGPS (Table 1) (Alatshan and Benkő, 2021; Coll-Bonfill et al., 2020; Krishnan and Feldman, 2011; Olson et al., 2017; Zhang et al., 2020). The fact that all these pathways are repressed by calcitriol suggests that the calcitriol/VDR axis regulates major upstream events in the inflammatory response.

Nuclear architectural abnormalities induced by progerin trigger the loss of VDR, similarly to the reduced levels of VDR in cells cultured for many passages “aging” in vitro. Studies using VDR agonist, calcitriol, restored expression of VDR in HGPS cells and improved several progeria hallmarks *in vitro* and *in vivo*. In HGPS cells, calcitriol mitigates misshapen cell nuclei, DNA damage, and DNA replication stress, contributing to the maintenance of nuclear structure and function (Cancado de Faria et al., 2023; Kreienkamp et al., 2018, 2016). Progerin-induced replication stress is characterized by a severe depletion of RAD51, a key genome safeguard, resulting in deprotection of stalled replication forks followed by degradation of newly synthesized DNA. Calcitriol not only elevates RAD51 levels, but also prevents nascent DNA resection at stalled replication forks, thereby restoring fork stability in progeria fibroblasts (Coll-Bonfill et al., 2020). These improvements in nuclear integrity and genome stability are followed by a suppression of the aberrant antiviral response in HGPS patient cells. Specifically, calcitriol reduces the expression of pro-inflammatory cytokines (*IL6, IL1B*, and *IFNB*), inhibits STAT1-mediated IFN response, and downregulates nearly 50 ISGs, highlighting its potent anti-inflammatory activity in HGPS (Cancado de Faria et al., 2023; Kreienkamp et al., 2018).

Inflammation, replication stress, and DNA damage have been identified as key contributors to VSMC loss in progeria. The expression of progerin promotes a phenotypic shift in VSMCs toward a synthetic/proliferative state, characterized by exacerbated DNA replication stress, increased DNA damage, and heightened cell death. This switch is accompanied by an upregulation of pro-inflammatory and pro-fibrotic gene signatures, which along with VSMC depletion, compromises the contractile properties of the aorta. Notably, calcitriol therapy prevents replication stress, DNA damage, and cell death in progerin-expressing VSMCs, underscoring the critical role of VDR agonism in mitigating vascular stiffness and degeneration in HGPS (Coll-Bonfill et al., 2023).

Moreover, the anti-inflammatory effects of calcitriol are linked to the amelioration of cell growth deficiencies and metabolic problems in progeria fibroblasts, particularly in autophagy and mitochondrial activity. Progerin disrupts autophagic flux and drives metabolic reprogramming in cells, favoring glycolysis and reducing mitochondrial oxidative phosphorylation (Cancado de Faria et al., 2023; Maynard et al., 2022; Rivera-Torres et al., 2013). Administration of calcitriol restores autophagic flux and improves mitochondrial respiration, bringing these processes closer to normal cellular function, thereby reducing cellular starvation and improving overall metabolic fitness. Importantly, *in vivo* studies further support the therapeutic potential of calcitriol, as its administration significantly delays premature death in progeria mice (Cancado de Faria et al., 2023). This suggests that calcitriol could serve as a promising therapeutic strategy for HGPS. However, despite its demonstrated impact on lifespan extension, further studies are necessary to assess its effects on tissue health, particularly on aortic abnormalities. Given the critical role of vascular complications in HGPS, the potential for calcitriol to remodel and improve aortic pathology remains an important area of investigation.

### 6.2. NLRP3 inhibition

The activation of NLRP3 inflammasome is reportedly detrimental for tissue degeneration during aging, and ablation of NLRP3 inflammasome extends longevity and health in aging mice, mitigating ovarian and vascular aging, atherosclerosis, bone loss, metabolic diseases, and neurodegeneration (Liang et al., 2024; Marín-Aguilar et al., 2020). In progeria, the role of NLRP3 was first investigated using MCC950 (Table 1), a small-molecule inhibitor that prevents NLRP3 from hydrolyzing ATP, thereby blocking inflammasome activation. MCC950 treatment reduced inflammasome activity while improving nuclear morphology and cell proliferation in HGPS fibroblasts. In a progeria mouse model (*Zmpste24*^−/−^), MCC950 administration lowered serum levels of IL-1β, increased body weight, and extended lifespan, highlighting the critical role of inflammasomes in disease progression (Coll et al., 2019; González-Dominguez et al., 2021). However, concerns over adverse effects and hepatotoxicity on healthy humans led to discontinuation of MCC950 in Phase II clinical trials, prompting researchers to explore alternative strategies. One such approach involves dapansutrile (OLT1177), a selective NLRP3 inhibitor that prevents inflammasome assembly, caspase-1 activation, and IL-1β maturation (Muela-Zarzuela et al., 2024). Unlike MCC950, dapansutrile has demonstrated safety in Phase I trials and is being investigated for inflammatory and cardiovascular diseases (Marchetti et al., 2018). In progeria models, dapansutrile reduced inflammation and cellular senescence, improved physiological features such as kyphosis, and extended lifespan (Table 1). Notably, when combined with lonafarnib, dapansutrile provided synergistic benefits in both fibroblasts and mouse models, supporting its potential as a promising dual-therapy approach for HGPS treatment (Muela-Zarzuela et al., 2024).

### 6.3. STING inhibition

Targeting the intracellular DNA sensing pathway through STING inhibition has gained significant attention as a promising therapeutic strategy across various disease contexts, leading to the development of multiple small-molecule inhibitors (Haag et al., 2018; Zhang and Zhang, 2025). The cGAS-STING pathway has recently been identified as a key mediator of chronic inflammation and functional decline associated with aging. Suppressing STING activity alleviates inflammatory responses in senescent human cells and tissues, reduces age-related inflammation in peripheral organs and the brain, and ultimately enhances overall tissue function in aging mice (Gulen et al., 2023). Likewise, in HGPS, targeting the STING pathway has demonstrated significant therapeutic benefits both *in vitro* and *in vivo*, despite its “non-canonical/alternative” behavior (Table 1 and Fig. 3). Our recent study utilized H-151, a potent and selective covalent STING antagonist that blocks STING palmitoylation and impairs its functionality. Either H-151 inhibitor or genetic depletion of STING effectively suppressed the expression of inflammatory cytokines and SASP in progeria fibroblasts, leading to improved proliferative capacity. *In vivo*, treatment with H-151 significantly extended the lifespan of progeria mice, promoting healthier aging without noticeable variations with sexual dimorphism. Moreover, STING inhibition helped maintain body weight and tissue integrity. In the cardiovascular system, H-151 treatment reduced VSMC loss and elastin fragmentation in the aortic arch, highlighting the detrimental role of STING activation in cardiovascular pathology, similar to its involvement in other cardiovascular diseases (An et al., 2024; Pham et al., 2021). Beyond its cardiovascular effects, H-151 therapy also demonstrated significant benefits in progeria-associated lipodystrophy by delaying fat loss, mitigating visceral lipoatrophy, and normalizing mitochondrial function in white adipocytes. While STING inhibition has been previously shown to be beneficial in obesity and fatty liver diseases (Bai et al., 2020; Hong et al., 2024; Luo et al., 2018; Yu et al., 2018), the positive impact of H-151 on progeria-related adipose tissue dysfunction suggests broader therapeutic potential for other forms of lipodystrophy, regardless of *LMNA* mutation status.

### 6.4. JAK-STAT blockage

The JAK-STAT pathway serves as a common road for signal transduction of different cytokines in cells, involving multiple interleukins and IFNs. Given its critical role in immune regulation, increasing evidence has linked dysregulated JAK-STAT signaling to autoimmune diseases, age-related disorders, neurodegenerative conditions, and malignancies. Consequently, this pathway has emerged as a promising therapeutic target (Hu et al., 2021; O’Shea et al., 2015). JAK-STAT inhibition strategies can be broadly categorized into three approaches: (1) blockage of cytokine or their receptor, (2) inhibition of JAK, and (3) antagonism of STATs. Currently, several JAK-STAT inhibitors are undergoing clinical trials for a range of diseases, with an expanding number of FDA-approved therapies. These include receptor-targeting agents such as tocilizumab (anti-IL-6 receptor) and anifrolumab (anti-IFN receptor), as well as JAK inhibitors such as tofacitinib, ruxolitinib, and baricitinib (Xue et al., 2023).

In HGPS, researchers have targeted IL-6-mediated JAK-STAT signaling using tocilizumab (Table 1), a monoclonal antibody commonly employed in the treatment of inflammatory diseases such as rheumatoid arthritis. Tocilizumab functions by neutralizing IL-6 through the inhibition of both soluble and membrane-bound IL-6 receptors (Scott, 2017; Squarzoni et al., 2021). In HGPS patient-derived cells, tocilizumab effectively blocked STAT3 activation, reduced progerin accumulation, and diminished DNA damage, ultimately attenuating cellular senescence. Consistently, IL-6 inhibition in a progeria mouse model (*Lmna*^*G609G/G609G*^) led to significant improvements in overall health, delaying morbidity and premature death. Enhanced motor function was accompanied by improvements in muscle, bone, and tendon integrity. Additionally, IL-6 blockade alleviated cardiovascular abnormalities, including aortic lesions and cardiac hypertrophy. Interestingly, tocilizumab also had beneficial effects on progeroid adipose tissue, promoting increased adipocyte size and adipogenesis (Squarzoni et al., 2021). While the potential synergistic effects of tocilizumab and lonafarnib remain to be explored, future studies are warranted to determine whether IL-6 inhibition could enhance the therapeutic benefits of lonafarnib in HGPS. Given its anti-inflammatory properties in aging, this study also highlights the potential application of tocilizumab in other laminopathies and age-related diseases.

Besides cytokine and receptor blockage, inhibition of the JAK-STAT pathway in HGPS has emerged as a promising therapeutic approach, with studies demonstrating the efficacy of JAK inhibitors such as baricitinib (Table 1). Research utilizing diverse cellular models, including HGPS patient-derived fibroblasts and isogenic dermal fibroblasts expressing progerin, has consistently shown that JAK-STAT inhibition significantly mitigates disease phenotypes (Cancado de Faria et al., 2023; Liu and Henriques, 2019). While patient-derived fibroblasts provide disease-relevant insights, they lack ideal isogenic controls, leading to some variability across studies. Conversely, isogenic progerin-expressing cells offer more controlled comparisons but may not fully capture the complexity of HGPS pathology. Nevertheless, findings across multiple models converge, strengthening the therapeutic potential of JAK-STAT blockage. Treatment of progeria cells with baricitinib led to a substantial reduction in the SASP, inflammatory cytokines, and IFN response at both transcriptional and protein levels. These anti-inflammatory effects were accompanied by improvements in hallmark cellular dysfunctions, including nuclear morphological abnormalities, DNA damage, mitochondrial dysfunction, oxidative stress, and autophagy deficiency, thereby reducing cell senescence and promoting cell proliferation (Arnold et al., 2021; Cancado de Faria et al., 2023; Liu and Henriques, 2019). Notably, the combination of baricitinib with lonafarnib was evaluated in patient-derived fibroblasts. While lonafarnib monotherapy alleviated several HGPS phenotypes, it paradoxically induced cytosolic DNA accumulation and hyperactivation of the cGAS-STING pathway, exacerbating inflammation and the IFN response. However, co-administration of baricitinib effectively mitigated these adverse effects, suggesting that JAK-STAT inhibition may counteract lonafarnib-induced toxicity and enhance therapeutic efficacy in HGPS patients (Arnold et al., 2021).

In alignment with *in vitro* findings, pharmacological JAK inhibition in progeria mouse models has demonstrated significant therapeutic benefits. Ruxolitinib treatment reduced premature aging phenotypes such as bone fractures, impaired bone mineralization, and muscle weakness in *Zmpste24*^−/−^ mice, with a trend toward increased survival (Table 1) (Griveau et al., 2020). Similarly, baricitinib administration in *Lmna*^*G609G/G609G*^ mice resulted in profound tissue improvements, leading to extended health span and lifespan (Cancado de Faria et al., 2023). Even in progeria mice maintained on a HFD regimen, where long-lived mice show the severity of the disease, baricitinib treatment conferred substantial benefits at both tissue and systemic levels, regardless of sex. JAK-STAT inhibition further enhanced muscle strength and prevented alopecia, a typical phenotype of HGPS. Beyond reducing hair loss, baricitinib restored dermal integrity by counteracting progerin-induced skin degeneration, implicating JAK-STAT signaling as a key contributor to skin pathologies in HGPS, consistent with its role in inflammatory skin disorders such as alopecia areata. In the cardiovascular system, baricitinib treatment prevented VSMC loss, an important factor in preventing arterial stiffness and vascular dysfunction in HGPS. Additionally, baricitinib effectively attenuated lipoatrophy in progeria mice by improving adipocyte size, reducing aberrant adipose tissue remodeling, and normalizing mitochondrial function in white adipose tissue, underscoring its potential role in treating progeria-associated lipodystrophy (Cancado de Faria et al., 2023). Although lonafarnib has demonstrated efficacy in addressing various tissue abnormalities, progeria-associated lipodystrophy remains an unresolved issue. The ability of baricitinib to enhance adipogenesis in HGPS pre-adipocytes, as well as in pre-adipocytes derived from other *LMNA*-associated lipodystrophies, suggests that JAK-STAT inhibition alone or in combination with lonafarnib may be expanded to laminopathic lipodystrophies (Hartinger et al., 2023; Najdi et al., 2021).

Finally, targeting STAT1 specifically in HGPS has yielded comparable outcomes to JAK-STAT inhibition (Table 1) (Cancado de Faria et al., 2023; Kreienkamp et al., 2018). Knockdown of STAT1 via short-hairpin RNA (shSTAT1) in progerin-expressing cells significantly reduced IFN signaling, improved cell proliferation, and enhanced cellular migration, which are key indicators of fibroblast health (Kreienkamp et al., 2018). *In vivo*, genetic ablation of one *Stat1* allele (*Stat1*^+/−^) in progeria mice (*Lmna*^*G609G/G609G*^) resulted in extended survival and improved tissue health, with phenotypic rescue observed in the skin, aorta, and adipose tissue. These findings underscore STAT1 as a pivotal mediator of IFN-driven inflammation, cellular and tissular dysfunction, and organismal aging in HGPS (Cancado de Faria et al., 2023). Future investigations would advance knowledge in the field by addressing the therapeutic potential of STAT1-targeting interventions for HGPS, aging-associated diseases, and other laminopathies.

## 7. Conclusions

The critical and beneficial role of inflammation in the protection of cells and tissues from the invasion of pathogens is indisputable. In recent years however, studies have shown the contribution of sterile inflammation (in the absence of pathogens) to the pathophysiology of a broad range of diseases, including cardiovascular, intestinal, neurological, and oncological diseases. In this review, we have highlighted what is currently known about sterile inflammation in a specific group of diseases caused by alterations of lamins, named laminopathies. There is strong evidence that alterations in lamins lead to disruption of the nuclear envelope and nuclear fragility, which combined with genomic instability (DNA repair and replication problems), and mitochondrial dysfunction, lead to the buildup of self-DNA in the cytoplasm. This self-DNA is recognized by different sensors, which are part of the inflammasome, the cGAS-STING pathway, or alternative/non-canonical pathways, and that trigger a plethora of inflammatory signaling cascades. A number of studies have demonstrated that targeting self-DNA sensing and signaling pathways can improve the phenotypes of laminopathies at a cellular, tissue, and organismal level. However, we currently have a very limited mechanistic understanding of how lamins dysfunction leads to self-DNA accumulation, how different cytosolic DNA species are recognized or discriminated by sensors, how sensors define the signaling through specific inflammatory cascades, and how the different inflammatory pathways impact cellular and tissue homeostasis.

## Figures and Tables

**Table 1:** Inflammatory targets and therapeutic benefits in HGPS models.

Target	Compound / Inhibitor	HGPS models	*In vitro* improvements	*In vivo* improvements	Approx. extended lifespan (%)	References
**STING**	H151	BJ, HDF fibroblasts. *Lmna*^*G609G/G609G*^ mice.	Proliferation, inflammation	Longevity, lipodystrophy Improved tissues: Aorta, white adipose tissue	Mixed - 26	
**IL–6**	Tocilizumab	HGPS fibroblasts *Lmna*^*G609G*/+^ preadipocytes. *Lmna*^*G609G*/+^, *Lmna*^*G609G/G609G*^ mice.	Proliferation, inflammation, nuclear shape, adipogenesis, DNA damage, senescence	Kyphosis, lethargy, body weight, Improved tissues: Aorta, white adipose tissue, skeletal and cardiac muscles, tendons, bone	*Lmna*^*G609G*/+^ Mixed – 36 *Lmna*^*G609G/G609G*^ Mixed - 13	(Squarzoni et al., 2021)
**JAK-STAT**	Ruxolitinib	MRC–5 fibroblasts. *Zmpste24*^−/−^ mice.	Proliferation, inflammation	Muscle strength, bone mineralization, longevity	Mixed - 11	(Griveau et al., 2020)
**JAK-STAT**	Baricitinib	HGPS, BJ, HDF fibroblasts, HGPS differentiated SKPs. *Lmna*^*G609G/G609G*^ mice.	Proliferation, inflammation, autophagy, oxidative stress, DNA damage, mitochondrial function, adipogenesis, nuclear shape, proteasome activity, senescence	Longevity, body weight, muscle strength, alopecia, lipodystrophy Improved tissues: Aorta, white adipose tissue, skin	*Baricitinib* Male – 13 *Baricitinib* + *HFD* Male - 47	(Arnold et al., 2021; Cancado de Faria et al., 2023; Hartinger et al., 2023; Liu and Henriques, 2019; Najdi et al., 2021)
**STAT1**	shSTAT1, *Statl*^+/−^	BJ fibroblasts. *Lmna*^*G609G/G609G*^ mice.	Proliferation, inflammation, migration	Body weight, muscle strength, lipodystrophy, longevity Improved tissues: Aorta, white adipose tissue, skin	Male - 19 Female - 18 Mixed - 15	(Cancado de Faria et al., 2023; Kreienkamp et al., 2018)
**NLRP3**	MCC950	HGPS fibroblasts. *Zmpste24*^*−*/-^, *Lmna*^*G609G/G609G*^ mice.	Proliferation, inflammation, nuclear morphology	Body weight, longevity, inflammation	Male - 19	(González-Dominguez et al., 2021)
**NLRP3**	Dapansutrile	HGPS fibroblasts *Lmna*^*G609G/G609G*^ mice.	Proliferation, inflammation, senescence	Kyphosis, body weight, inflammation, longevity Improved tissues: heart and liver	Male - 40	(Muela-Zarzuela et al., 2024)
**Multiple**	Calcitriol	HGPS, BJ, HDF fibroblasts. *Lmna*^*G609G/G609G*^ mice.	Proliferation, inflammation, nuclear morphology, DNA damage, DNA replication, differentiation, mitochondrial function, autophagy, anti-viral response	Morbidity, longevity	Female - 22	(Cancado de Faria et al., 2023; Coll-Bonfill et al., 2023; Coll-Bonfill et al., 2020; Kreienkamp et al., 2018, 2016)

## Data Availability

Data will be made available on request.

## References

[R1] Ahn J, Jo I, Kang S, Hong S, Kim S, Jeong S, Kim Y-H, Park B-J, Ha N-C, 2019. Structural basis for lamin assembly at the molecular level. Nat. Commun 10, 3757. 10.1038/s41467-019-11684-x. 31434876 PMC6704074

[R2] Akinci B, Swaidan M, Foss-Freitas MC, Luo Y, Neidert AH, Hench RP, Chenevert TL, Longcore A, Bakker-Arkema R, Bisgaier CL, Oral EA, 2020. 2214-PUB: an Open-Label study of gemcabene in adults with familial partial lipodystrophy. Diabetes 69. 10.2337/db20-2214-PUB.

[R3] Al Tanoury Z, Zimmerman JF, Rao J, Sieiro D, McNamara HM, Cherrier T, Rodríguez-delaRosa A, Hick-Colin A, Bousson F, Fugier-Schmucker C, Marchiano F, Habermann B, Chal J, Nesmith AP, Gapon S, Wagner E, Gupta VA, Bassel-Duby R, Olson EN, Cohen AE, Parker KK, Pourquié O, 2021. Prednisolone rescues duchenne muscular dystrophy phenotypes in human pluripotent stem cell–derived skeletal muscle in vitro. Proc. Natl. Acad. Sci 118. 10.1073/pnas.2022960118. PMC8285911 34260377

[R4] Alatshan A, Benkő S, 2021. Nuclear receptors as multiple regulators of NLRP3 inflammasome function. Front Immunol. 12. 10.3389/fimmu.2021.630569. PMC7952630 33717162

[R5] An C, Li Z, Chen Y, Huang S, Yang F, Hu Y, Xu T, Zhang C, Ge S, 2024. The cGAS-STING pathway in cardiovascular diseases: from basic research to clinical perspectives. Cell Biosci. 14, 58. 10.1186/s13578-024-01242-4. 38720328 PMC11080250

[R6] Arnold R, Vehns E, Randl H, Djabali K, 2021. Baricitinib, a JAK-STAT inhibitor, reduces the cellular toxicity of the farnesyltransferase inhibitor lonafarnib in progeria cells. Int J. Mol. Sci 22, 7474. 10.3390/ijms22147474. 34299092 PMC8307450

[R7] Atchison L, Abutaleb NO, Snyder-Mounts E, Gete Y, Ladha A, Ribar T, Cao K, Truskey GA, 2020. iPSC-Derived endothelial cells affect vascular function in a Tissue-Engineered blood vessel model of Hutchinson-Gilford progeria syndrome. Stem Cell Rep. 14, 325–337. 10.1016/j.stemcr.2020.01.005. PMC7013250 32032552

[R8] Bai J, Cervantes C, He S, He J, Plasko GR, Wen J, Li Z, Yin D, Zhang C, Liu M, Dong LQ, Liu F, 2020. Mitochondrial stress-activated cGAS-STING pathway inhibits thermogenic program and contributes to overnutrition-induced obesity in mice. Commun. Biol 3, 257. 10.1038/s42003-020-0986-1. 32444826 PMC7244732

[R9] Balahura LR, Selaru A, Dinescu S, Costache M, 2020. Inflammation and inflammasomes: pros and cons in tumorigenesis. J. Immunol. Res 2020, 1–15. 10.1155/2020/2549763. PMC7520695 33015196

[R10] Bernasconi P, 2015. Altered cytokine profiles in laminopathic patients. Orphanet J. Rare Dis 10, O14. 10.1186/1750-1172-10-S2-O14.

[R11] Brayson D, Frustaci A, Verardo R, Chimenti C, Russo MA, Hayward R, Ahmad SM, Vizcay-Barrena G, Protti A, Zammit PS, Remedios dos CG, Ehler E, Shah AM, Shanahan CM, 2019. Prelamin a mediates myocardial inflammation in dilated and HIV-associated cardiomyopathies. JCI Insight 4. 10.1172/jci.insight.126315. PMC6948859 31622279

[R12] Burleigh K, Maltbaek JH, Cambier S, Green R, Gale M, James RC, Stetson DB, 2020. Human DNA-PK activates a STING-independent DNA sensing pathway. Sci. Immunol 5. 10.1126/sciimmunol.aba4219. PMC7081723 31980485

[R13] Cabral A, Cabral JE, Wang A, Zhang Y, Liang H, Nikbakht D, Corona L, Hoffman HM, McNulty R, 2023. Differential binding of NLRP3 to non-oxidized and Ox-mtDNA mediates NLRP3 inflammasome activation. Commun. Biol 6, 578. 10.1038/s42003-023-04817-y. 37253813 PMC10229695

[R14] Cancado de Faria R, Shashkova EV, Flaveny C, Baldan A, McCommis KS, Gonzalo S, 2023. STAT1 drives the Interferon-Like response and aging hallmarks in progeria. Aging Biol. 1, 20230009. 10.59368/agingbio.20230009. PMC12007894 40255266

[R15] Cao K, Graziotto JJ, Blair CD, Mazzulli JR, Erdos MR, Krainc D, Collins FS, 2011. Rapamycin reverses cellular phenotypes and enhances mutant protein clearance in Hutchinson-Gilford progeria syndrome cells. Sci. Transl. Med 3. 10.1126/scitranslmed.3002346. 21715679

[R16] Cappelletti C, Salerno F, Canioni E, Mora M, Mantegazza R, Bernasconi P, Maggi L, 2018. Up-regulation of Toll-like receptors 7 and 9 and its potential implications in the pathogenic mechanisms of *lmna* -related myopathies. Nucleus 9, 398–409. 10.1080/19491034.2018.1471947. 29895224 PMC7000140

[R17] Cappelletti C, Tramacere I, Cavalcante P, Schena E, Politano L, Carboni N, Gambineri A, D’Amico A, Ruggiero L, Ricci G, Siciliano G, Boriani G, Mongini TE, Vercelli L, Biagini E, Ziacchi M, D’Apice MR, Lattanzi G, Mantegazza R, Maggi L, Bernasconi P, 2020. Cytokine profile in striated muscle laminopathies: new promising biomarkers for disease prediction. Cells 9, 1532. 10.3390/cells9061532. 32585971 PMC7348753

[R18] Chatzidoukaki O, Stratigi K, Goulielmaki E, Niotis G, Akalestou-Clocher A, Gkirtzimanaki K, Zafeiropoulos A, Altmüller J, Topalis P, Garinis GA, 2021. R-loops trigger the release of cytoplasmic ssDNAs leading to chronic inflammation upon DNA damage. Sci. Adv 7. 10.1126/sciadv.abj5769. PMC8604417 34797720

[R19] Cheedipudi SM, Asghar S, Marian AJ, 2022. Genetic ablation of the DNA damage response pathway attenuates Lamin-Associated dilated cardiomyopathy in mice. JACC Basic Transl. Sci 7, 1232–1245. 10.1016/j.jacbts.2022.06.015. 36644279 PMC9831927

[R20] Chen GY, Nuñez G, 2010. Sterile inflammation: sensing and reacting to damage. Nat. Rev. Immunol 10, 826–837. 10.1038/nri2873. 21088683 PMC3114424

[R21] Chen Q, Boire A, Jin X, Valiente M, Er EE, Lopez-Soto A, S. Jacob L, Patwa R, Shah H, Xu K, Cross JR, Massagué J, 2016. Carcinoma–astrocyte gap junctions promote brain metastasis by cGAMP transfer. Nature 533, 493–498. 10.1038/nature18268. 27225120 PMC5021195

[R22] Coll RC, Hill JR, Day CJ, Zamoshnikova A, Boucher D, Massey NL, Chitty JL, Fraser JA, Jennings MP, Robertson AAB, Schroder K, 2019. MCC950 directly targets the NLRP3 ATP-hydrolysis motif for inflammasome inhibition. Nat. Chem. Biol 15, 556–559. 10.1038/s41589-019-0277-7. 31086327

[R23] Coll-Bonfill N, Mahajan U, Shashkova EV, Lin C-J, Mecham RP, Gonzalo S, 2023. Progerin induces a phenotypic switch in vascular smooth muscle cells and triggers replication stress and an aging-associated secretory signature. Geroscience 45, 965–982. 10.1007/s11357-022-00694-1. 36482259 PMC9886737

[R24] Coll-Bonfill N, Cancado de Faria R, Bhoopatiraju S, Gonzalo S, 2020. Calcitriol prevents RAD51 loss and cGAS-STING-IFN response triggered by progerin. Proteomics 20, 1800406. 10.1002/pmic.201800406. PMC7117971 31834988

[R25] Colotta F, Jansson B, Bonelli F, 2017. Modulation of inflammatory and immune responses by vitamin d. J. Autoimmun 85, 78–97. 10.1016/j.jaut.2017.07.007. 28733125

[R26] Di Micco A, Frera G, Lugrin J, Jamilloux Y, Hsu E-T, Tardivel A, De Gassart A, Zaffalon L, Bujisic B, Siegert S, Quadroni M, Broz P, Henry T, Hrycyna CA, Martinon F, 2016. AIM2 inflammasome is activated by pharmacological disruption of nuclear envelope integrity. Proc. Natl. Acad. Sci 113. 10.1073/pnas.1602419113. PMC4987819 27462105

[R27] Dinarello CA, 2009. Immunological and inflammatory functions of the Interleukin-1 family. Annu Rev. Immunol 27, 519–550. 10.1146/annurev.immunol.021908.132612. 19302047

[R28] Dinarello CA, Simon A, van der Meer JWM, 2012. Treating inflammation by blocking interleukin-1 in a broad spectrum of diseases. Nat. Rev. Drug Discov 11, 633–652. 10.1038/nrd3800. 22850787 PMC3644509

[R29] Dixon CR, Malik P, de las Heras JI, Saiz-Ros N, de Lima Alves F, Tingey M, Gaunt E, Richardson AC, Kelly DA, Goldberg MW, Towers GJ, Yang W, Rappsilber J, Digard P, Schirmer EC, 2021. STING nuclear partners contribute to innate immune signaling responses. iScience 24, 103055. 10.1016/j.isci.2021.103055. 34541469 PMC8436130

[R30] Dobrzynska A, Gonzalo S, Shanahan C, Askjaer P, 2016. The nuclear lamina in health and disease. Nucleus 7, 233–248. 10.1080/19491034.2016.1183848. PMC4991244 27158763

[R31] Dong M, Fitzgerald KA, 2024. DNA-sensing pathways in health, autoinflammatory and autoimmune diseases. Nat. Immunol 25, 2001–2014. 10.1038/s41590-024-01966-y. 39367124

[R32] Dunphy G, Flannery SM, Almine JF, Connolly DJ, Paulus C, Jønsson KL, Jakobsen MR, Nevels MM, Bowie AG, Unterholzner L, 2018. Non-canonical activation of the DNA sensing adaptor STING by ATM and IFI16 mediates NF-κB signaling after nuclear DNA damage. Mol. Cell 71, 745–760.e5. 10.1016/j.molcel.2018.07.034. 30193098 PMC6127031

[R33] Dvorkin S, Cambier S, Volkman HE, Stetson DB, 2024. New frontiers in the cGAS-STING intracellular DNA-sensing pathway. Immunity 57, 718–730. 10.1016/j.immuni.2024.02.019. 38599167 PMC11013568

[R34] Earle AJ, Kirby TJ, Fedorchak GR, Isermann P, Patel J, Iruvanti S, Moore SA, Bonne G, Wallrath LL, Lammerding J, 2020. Mutant lamins cause nuclear envelope rupture and DNA damage in skeletal muscle cells. Nat. Mater 19, 464–473. 10.1038/s41563-019-0563-5. 31844279 PMC7102937

[R35] En A, Bogireddi H, Thomas B, Stutzman AV, Ikegami S, LaForest B, Almakki O, Pytel P, Moskowitz IP, Ikegami K, 2024. Pervasive nuclear envelope ruptures precede ECM signaling and disease onset without activating cGAS-STING in Lamin-cardiomyopathy mice. Cell Rep. 43, 114284. 10.1016/j.celrep.2024.114284. 38814785 PMC11290591

[R36] Ferguson BJ, Mansur DS, Peters NE, Ren H, Smith GL, 2012. DNA-PK is a DNA sensor for IRF-3-dependent innate immunity. Elife 1. 10.7554/eLife.00047. PMC3524801 23251783

[R37] Fong LG, Vickers TA, Farber EA, Choi C, Yun UJ, Hu Y, Yang SH, Coffinier C, Lee R, Yin L, Davies BSJ, Andres DA, Spielmann HP, Bennett CF, Young SG, 2009. Activating the synthesis of progerin, the mutant prelamin a in Hutchinson–Gilford progeria syndrome, with antisense oligonucleotides. Hum. Mol. Genet 18, 2462–2471. 10.1093/hmg/ddp184. 19376814 PMC2694694

[R38] Foss-Freitas MC, Ferraz RC, Monteiro LZ, Gomes PM, Iwakura R, de Freitas LCC, Foss MC, 2018. Endoplasmic reticulum stress activation in adipose tissue induces metabolic syndrome in individuals with familial partial lipodystrophy of the dunnigan type. Diabetol. Metab. Syndr 10, 6. 10.1186/s13098-017-0301-6. 29449893 PMC5807843

[R39] Gabriel D, Roedl D, Gordon LB, Djabali K, 2015. Sulforaphane enhances progerin clearance in Hutchinson-Gilford progeria fibroblasts. Aging Cell 14, 78–91. 10.1111/acel.12300. 25510262 PMC4326906

[R40] Geng K, Ma X, Jiang Z, Huang W, Gu J, Wang P, Luo L, Xu Youhua, Xu Yong, 2023. High glucose-induced STING activation inhibits diabetic wound healing through promoting M1 polarization of macrophages. Cell Death Discov. 9, 136. 10.1038/s41420-023-01425-x. 37100799 PMC10133226

[R41] Gerbino A, Forleo C, Milano S, Piccapane F, Procino G, Pepe M, Piccolo M, Guida P, Resta N, Favale S, Svelto M, Carmosino M, 2021. Pro-inflammatory cytokines as emerging molecular determinants in cardiolaminopathies. J. Cell Mol. Med 25, 10902–10915. 10.1111/jcmm.16975. 34773379 PMC8642682

[R42] Gong T, Liu L, Jiang W, Zhou R, 2020. DAMP-sensing receptors in sterile inflammation and inflammatory diseases. Nat. Rev. Immunol 20, 95–112. 10.1038/s41577-019-0215-7. 31558839

[R43] González-Dominguez A, Montañez R, Castejón-Vega B, Nuñez-Vasco J, Lendines-Cordero D, Wang C, Mbalaviele G, Navarro-Pando JM, Alcocer-Gómez E, Cordero MD, 2021. Inhibition of the NLRP3 inflammasome improves lifespan in animal murine model of Hutchinson–Gilford progeria. EMBO Mol. Med 13. 10.15252/emmm.202114012. PMC8495449 34448355

[R44] Gonzalez-Suarez I, Redwood AB, Perkins SM, Vermolen B, Lichtensztejin D, Grotsky DA, Morgado-Palacin L, Gapud EJ, Sleckman BP, Sullivan T, Sage J, Stewart CL, Mai S, Gonzalo S, 2009. Novel roles for A-type lamins in telomere biology and the DNA damage response pathway. EMBO J. 28, 2414–2427. 10.1038/emboj.2009.196. 19629036 PMC2735177

[R45] Gonzalo S, Kreienkamp R, Askjaer P, 2017. Hutchinson-Gilford progeria syndrome: a premature aging disease caused by LMNA gene mutations. Ageing Res Rev. 33, 18–29. 10.1016/j.arr.2016.06.007. 27374873 PMC5195863

[R46] Graziano S, Kreienkamp R, Coll-Bonfill Nuria, Gonzalo S, 2018. Causes and consequences of genomic instability in laminopathies: replication stress and interferon response. Nucleus 9, 289–306. . 10.1080/19491034.2018.1454168 PMC5973265 29637811

[R47] Graziano S, Coll-Bonfill N, Teodoro-Castro B, Kuppa S, Jackson J, Shashkova E, Mahajan U, Vindigni A, Antony E, Gonzalo S, 2021. Lamin A/C recruits ssDNA protective proteins RPA and RAD51 to stalled replication forks to maintain fork stability. J. Biol. Chem 297, 101301. 10.1016/j.jbc.2021.101301. 34648766 PMC8571089

[R48] Griveau A, Wiel C, Ziegler DV, Bergo MO, Bernard D, 2020. The JAK1/2 inhibitor ruxolitinib delays premature aging phenotypes. Aging Cell 19. 10.1111/acel.13122. PMC7189991 32196928

[R49] Guglieri M, Bushby K, McDermott MP, Hart KA, Tawil R, Martens WB, Herr BE, McColl E, Speed C, Wilkinson J, Kirschner J, King WM, Eagle M, Brown MW, Willis T, Griggs RC, Straub V, van Ruiten H, Childs A-M, Ciafaloni E, Shieh PB, Spinty S, Maggi L, Baranello G, Butterfield RJ, Horrocks IA, Roper H, Alhaswani Z, Flanigan KM, Kuntz NL, Manzur A, Darras BT, Kang PB, Morrison L, Krzesniak-Swinarska M, Mah JK, Mongini TE, Ricci F, von der Hagen M, Finkel RS, O’Reardon K, Wicklund M, Kumar A, McDonald CM, Han JJ, Joyce N, Henricson EK, Schara-Schmidt U, Gangfuss A, Wilichowski E, Barohn RJ, Statland JM, Campbell C, Vita G, Vita GL, Howard JF, Hughes I, McMillan HJ, Pegoraro E, Bello L, Burnette WB, Thangarajh M, Chang T, 2022. Effect of different corticosteroid dosing regimens on clinical outcomes in boys with duchenne muscular dystrophy. JAMA 327, 1456. 10.1001/jama.2022.4315. 35381069 PMC8984930

[R50] Gulen MF, Samson N, Keller A, Schwabenland M, Liu C, Glück S, Thacker VV, Favre L, Mangeat B, Kroese LJ, Krimpenfort P, Prinz M, Ablasser A, 2023. cGAS–STING drives ageing-related inflammation and neurodegeneration. Nature 620, 374–380. 10.1038/s41586-023-06373-1. 37532932 PMC10412454

[R51] Haag SM, Gulen MF, Reymond L, Gibelin A, Abrami L, Decout A, Heymann M, van der Goot FG, Turcatti G, Behrendt R, Ablasser A, 2018. Targeting STING with covalent small-molecule inhibitors. Nature 559, 269–273. 10.1038/s41586-018-0287-8. 29973723

[R52] Hamczyk MR, del Campo L, Andrés V, 2018. Aging in the cardiovascular system: lessons from Hutchinson-Gilford progeria syndrome. Annu Rev. Physiol 80, 27–48. 10.1146/annurev-physiol-021317-121454. 28934587

[R53] Hartinger R, Lederer E-M, Schena E, Lattanzi G, Djabali K, 2023. Impact of combined baricitinib and FTI treatment on adipogenesis in Hutchinson–Gilford progeria syndrome and other lipodystrophic laminopathies. Cells 12, 1350. 10.3390/cells12101350. 37408186 PMC10216179

[R54] Herbelet S, De Paepe B, De Bleecker JL, 2020. Description of a novel mechanism possibly explaining the antiproliferative properties of glucocorticoids in duchenne muscular dystrophy fibroblasts based on glucocorticoid receptor GR and NFAT5. Int J. Mol. Sci 21, 9225. 10.3390/ijms21239225. 33287327 PMC7731298

[R55] Hodes RJ, Sierra F, Austad SN, Epel E, Neigh GN, Erlandson KM, Schafer MJ, LeBrasseur NK, Wiley C, Campisi J, Sehl ME, Scalia R, Eguchi S, Kasinath BS, Halter JB, Cohen HJ, Demark-Wahnefried W, Ahles TA, Barzilai N, Hurria A, Hunt PW, 2016. Disease drivers of aging. Ann. N. Y Acad. Sci 1386, 45–68. 10.1111/nyas.13299. PMC5373660 27943360

[R56] Hong Z, Chen S, Sun J, Cheng D, Guo H, Mei J, Zhang X, Maimaiti M, Hao H, Cao P, Hu H, Wang C, 2024. STING signaling in islet macrophages impairs insulin secretion in obesity. Sci. China Life Sci 67, 345–359. 10.1007/s11427-022-2371-9. 37906411

[R57] Hu X, Li J, Fu M, Zhao X, Wang W, 2021. The JAK/STAT signaling pathway: from bench to clinic. Signal Transduct. Target Ther 6, 402. 10.1038/s41392-021-00791-1. 34824210 PMC8617206

[R58] Huang Y, Li X, Luo G, Wang J, Li R, Zhou C, Wan T, Yang F, 2022. Pyroptosis as a candidate therapeutic target for Alzheimer’s disease. Front Aging Neurosci. 14. 10.3389/fnagi.2022.996646. PMC9520296 36185484

[R59] Hwang I, Uchida H, Dai Z, Li F, Sanchez T, Locasale JW, Cantley LC, Zheng H, Paik J, 2021. Cellular stress signaling activates type-I IFN response through FOXO3-regulated lamin posttranslational modification. Nat. Commun 12, 640. 10.1038/s41467-020-20839-0. PMC7843645 33510167

[R60] Kang S, Tanaka T, Narazaki M, Kishimoto T, 2019. Targeting Interleukin-6 signaling in clinic. Immunity 50, 1007–1023. 10.1016/j.immuni.2019.03.026. 30995492

[R61] Koblan LW, Erdos MR, Wilson C, Cabral WA, Levy JM, Xiong Z-M, Tavarez UL, Davison LM, Gete YG, Mao X, Newby GA, Doherty SP, Narisu N, Sheng Q, Krilow C, Lin CY, Gordon LB, Cao K, Collins FS, Brown JD, Liu DR, 2021b. In vivo base editing rescues Hutchinson–Gilford progeria syndrome in mice. Nature 589, 608–614. 10.1038/s41586-020-03086-7. 33408413 PMC7872200

[R62] Koblan LW, Erdos MR, Wilson C, Cabral WA, Levy JM, Xiong Z-M, Tavarez UL, Davison LM, Gete YG, Mao X, Newby GA, Doherty SP, Narisu N, Sheng Q, Krilow C, Lin CY, Gordon LB, Cao K, Collins FS, Brown JD, Liu DR, 2021a. In vivo base editing rescues Hutchinson–Gilford progeria syndrome in mice. Nature 589, 608–614. 10.1038/s41586-020-03086-7. 33408413 PMC7872200

[R63] Kreienkamp R, Croke M, Neumann MA, Bedia-Diaz G, Graziano S, Dusso A, Dorsett D, Carlberg C, Gonzalo S, 2016. Vitamin d receptor signaling improves Hutchinson-Gilford progeria syndrome cellular phenotypes. Oncotarget 7, 30018–30031. 10.18632/oncotarget.9065. 27145372 PMC5058660

[R64] Kreienkamp R, Graziano S, Coll-Bonfill N, Bedia-Diaz G, Cybulla E, Vindigni A, Dorsett D, Kubben N, Batista LFZ, Gonzalo S, 2018. A Cell-Intrinsic Interferon-like response links replication stress to cellular aging caused by progerin. Cell Rep. 22, 2006–2015. 10.1016/j.celrep.2018.01.090. 29466729 PMC5848491

[R65] Kreienkamp R, Billon C, Bedia-Diaz G, Albert CJ, Toth Z, Butler AA, McBride-Gagyi S, Ford DA, Baldan A, Burris TP, Gonzalo S, 2019. Doubled lifespan and patient-like pathologies in progeria mice fed high-fat diet. Aging Cell 18. 10.1111/acel.12852. PMC6351834 30548460

[R66] Krishnan AV, Feldman D, 2011. Mechanisms of the Anti-Cancer and Anti-Inflammatory actions of vitamin d. Annu Rev. Pharm. Toxicol 51, 311–336. 10.1146/annurev-pharmtox-010510-100611. 20936945

[R67] Krüger P, Hartinger R, Djabali K, 2024a. Navigating lipodystrophy: insights from laminopathies and beyond. Int J. Mol. Sci 25, 8020. 10.3390/ijms25158020. PMC11311807 39125589

[R68] Krüger P, Schroll M, Fenzl F, Lederer E-M, Hartinger R, Arnold R, Cagla Togan D, Guo R, Liu S, Petry A, Görlach A, Djabali K, 2024b. Inflammation and fibrosis in progeria: Organ-Specific responses in an HGPS mouse model. Int J. Mol. Sci 25, 9323. 10.3390/ijms25179323. 39273272 PMC11395088

[R69] Kubben N, Zhang W, Wang L, Voss TC, Yang J, Qu J, Liu G-H, Misteli T, 2016. Repression of the antioxidant NRF2 pathway in premature aging. Cell 165, 1361–1374. 10.1016/j.cell.2016.05.017. 27259148 PMC4893198

[R70] Lei Y, Guerra Martinez C, Torres-Odio S, Bell SL, Birdwell CE, Bryant JD, Tong CW, Watson RO, West LC, West AP, 2021. Elevated type I interferon responses potentiate metabolic dysfunction, inflammation, and accelerated aging in mtDNA mutator mice. Sci. Adv 7. 10.1126/sciadv.abe7548. PMC8153723 34039599

[R71] Li X, Li C, Zhang W, Wang Y, Qian P, Huang H, 2023. Inflammation and aging: signaling pathways and intervention therapies. Signal Transduct. Target Ther 8, 239. 10.1038/s41392-023-01502-8. PMC10248351 37291105

[R72] Li Y, Cui J, Liu L, Hambright WS, Gan Y, Zhang Y, Ren S, Yue X, Shao L, Cui Y, Huard J, Mu Y, Yao Q, Mu X, 2024. mtDNA release promotes cGAS-STING activation and accelerated aging of postmitotic muscle cells. Cell Death Dis. 15, 523. 10.1038/s41419-024-06863-8. 39039044 PMC11263593

[R73] Liang R, Qi X, Cai Q, Niu L, Huang X, Zhang D, Ling J, Wu Y, Chen Y, Yang P, Liu J, Zhang J, Yu P, 2024. The role of NLRP3 inflammasome in aging and age-related diseases. Immun. Ageing 21, 14. 10.1186/s12979-023-00395-z. PMC10840156 38317229

[R74] Liu Arnold, Henriques Djabali, 2019. Inhibition of JAK-STAT signaling with baricitinib reduces inflammation and improves cellular homeostasis in progeria cells. Cells 8, 1276. 10.3390/cells8101276. 31635416 PMC6829898

[R75] Liu W, Wang S, Zhang X, Ke Z, Wen X, Zhao J, Zhuang X, Liao L, 2024. Enhanced cardiomyocyte NLRP3 Inflammasome-Mediated pyroptosis promotes d -Galactose–Induced cardiac aging. J. Am. Heart Assoc 13. 10.1161/JAHA.123.032904. PMC11292767 38979831

[R76] Lugrin J, Martinon F, 2018. The AIM 2 inflammasome: sensor of pathogens and cellular perturbations. Immunol. Rev 281, 99–114. 10.1111/imr.12618. 29247998

[R77] Luo W, Zou X, Wang Y, Dong Z, Weng X, Pei Z, Song S, Zhao Y, Wei Z, Gao R, Zhang B, Liu L, Bai P, Liu J, Wang X, Gao T, Zhang Y, Sun X, Chen H, Hu K, Du S, Sun A, Ge J, 2023. Critical role of the cGAS-STING pathway in Doxorubicin-Induced cardiotoxicity. Circ. Res 132. 10.1161/CIRCRESAHA.122.321587. 37154056

[R78] Luo X, Li H, Ma L, Zhou J, Guo X, Woo S-L, Pei Y, Knight LR, Deveau M, Chen Y, Qian X, Xiao X, Li Q, Chen X, Huo Y, McDaniel K, Francis H, Glaser S, Meng F, Alpini G, Wu C, 2018. Expression of STING is increased in liver tissues from patients with NAFLD and promotes Macrophage-Mediated hepatic inflammation and fibrosis in mice. Gastroenterology 155, 1971–1984.e4. 10.1053/j.gastro.2018.09.010. 30213555 PMC6279491

[R79] MacDonald KM, Nicholson-Puthenveedu S, Tageldein MM, Khasnis S, Arrowsmith CH, Harding SM, 2023. Antecedent chromatin organization determines cGAS recruitment to ruptured micronuclei. Nat. Commun 14, 556. 10.1038/s41467-023-36195-8. 36732527 PMC9894866

[R80] MacRae CA, Taylor MRG, Mestroni L, Moses J, Ashley EA, Wheeler MT, Lakdawala NK, Hershberger RE, Sandor V, Saunders ME, Oliver C, Lee PA, Judge DP, 2023. Efficacy and safety of ARRY-371797 in *lmna* -Related dilated cardiomyopathy: a phase 2 study. Circ. Genom. Precis Med 16. 10.1161/CIRCGEN.122.003730. PMC9946172 36515663

[R81] Marchetti C, Swartzwelter B, Gamboni F, Neff CP, Richter K, Azam T, Carta S, Tengesdal I, Nemkov T, D’Alessandro A, Henry C, Jones GS, Goodrich SA, St. Laurent JP, Jones TM, Scribner CL, Barrow RB, Altman RD, Skouras DB, Gattorno M, Grau V, Janciauskiene S, Rubartelli A, Joosten LAB, Dinarello CA, 2018. OLT1177, a β-sulfonyl nitrile compound, safe in humans, inhibits the NLRP3 inflammasome and reverses the metabolic cost of inflammation. Proc. Natl. Acad. Sci 115. 10.1073/pnas.1716095115. PMC5816172 29378952

[R82] Marín-Aguilar F, Lechuga-Vieco AV, Alcocer-Gómez E, Castejón-Vega B, Lucas J, Garrido C, Peralta-Garcia A, Pérez-Pulido AJ, Varela-López A, Quiles JL, Ryffel B, Flores I, Bullón P, Ruiz-Cabello J, Cordero MD, 2020. NLRP3 inflammasome suppression improves longevity and prevents cardiac aging in Male mice. Aging Cell 19. 10.1111/acel.13050. PMC6974709 31625260

[R83] Mathis D, 2024. Hunting down the elusive cytosolic-DNA sensor. Proc. Natl. Acad. Sci 121. 10.1073/pnas.2415648121. PMC11441527 39297679

[R84] Maynard S, Hall A, Galanos P, Rizza S, Yamamoto T, Gram HH, Munk SHN, Shoaib M, Sørensen CS, Bohr VA, Lerdrup M, Maya-Mendoza A, Bartek J, 2022. Lamin A/C impairments cause mitochondrial dysfunction by attenuating PGC1α and the NAMPT-NAD+ pathway. Nucleic Acids Res 50, 9948–9965. 10.1093/nar/gkac741. 36099415 PMC9508839

[R85] McNab F, Mayer-Barber K, Sher A, Wack A, O’Garra A, 2015. Type I interferons in infectious disease. Nat. Rev. Immunol 15, 87–103. 10.1038/nri3787. 25614319 PMC7162685

[R86] Mehl JL, Earle A, Lammerding J, Mhlanga M, Vogel V, Jain N, 2022. Blockage of lamin-A/C loss diminishes the pro-inflammatory macrophage response. iScience 25, 105528. 10.1016/j.isci.2022.105528. 36465100 PMC9708799

[R87] Miller KN, Victorelli SG, Salmonowicz H, Dasgupta N, Liu T, Passos JF, Adams PD, 2021. Cytoplasmic DNA: sources, sensing, and role in aging and disease. Cell 184, 5506–5526. 10.1016/j.cell.2021.09.034. 34715021 PMC8627867

[R88] Mu X, Tseng C, Hambright WS, Matre P, Lin C, Chanda P, Chen W, Gu J, Ravuri S, Cui Y, Zhong L, Cooke JP, Niedernhofer LJ, Robbins PD, Huard J, 2020. Cytoskeleton stiffness regulates cellular senescence and innate immune response in Hutchinson–Gilford progeria syndrome. Aging Cell 19. 10.1111/acel.13152. PMC7431831 32710480

[R89] Muela-Zarzuela I, Suarez-Rivero JM, Boy-Ruiz D, López-Pérez J, Sotelo-Montoro M, del Mar Navarrete-Alonso M, Collado IG, Botubol-Ares JM, Sanz A, Cordero MD, 2024. The NLRP3 inhibitor dapansutrile improves the therapeutic action of lonafarnib on progeroid mice. Aging Cell 23. 10.1111/acel.14272. PMC11488313 39192596

[R90] Murtada S-I, Mikush N, Wang M, Ren P, Kawamura Y, Ramachandra AB, Li DS, Braddock DT, Tellides G, Gordon LB, Humphrey JD, 2023. Lonafarnib improves cardiovascular function and survival in a mouse model of Hutchinson-Gilford progeria syndrome. Elife 12. 10.7554/eLife.82728. PMC10023154 36930696

[R91] Najdi F, Krüger P, Djabali K, 2021. Impact of progerin expression on adipogenesis in Hutchinson—Gilford progeria Skin-Derived precursor cells. Cells 10, 1598. 10.3390/cells10071598. 34202258 PMC8306773

[R92] O’Shea JJ, Schwartz DM, Villarino AV, Gadina M, McInnes IB, Laurence A, 2015. The JAK-STAT pathway: impact on human disease and therapeutic intervention. Annu Rev. Med 66, 311–328. 10.1146/annurev-med-051113-024537. 25587654 PMC5634336

[R93] Olive M, Harten I, Mitchell R, Beers JK, Djabali K, Cao K, Erdos MR, Blair C, Funke B, Smoot L, Gerhard-Herman M, Machan JT, Kutys R, Virmani R, Collins FS, Wight TN, Nabel EG, Gordon LB, 2010. Cardiovascular pathology in Hutchinson-Gilford progeria: correlation with the vascular pathology of aging. Arterioscler. Thromb. Vasc. Biol 30, 2301–2309. 10.1161/ATVBAHA.110.209460. 20798379 PMC2965471

[R94] Olson KC, Kulling PM, Olson TL, Tan S-F, Rainbow RJ, Feith DJ, Loughran TP, 2017. Vitamin d decreases STAT phosphorylation and inflammatory cytokine output in T-LGL leukemia. Cancer Biol. Ther 18, 290–303. 10.1080/15384047.2016.1235669. 27715403 PMC5499847

[R95] Ortega-Gómez A, Perretti M, Soehnlein O, 2013. Resolution of inflammation: an integrated view. EMBO Mol. Med 5, 661–674. 10.1002/emmm.201202382. 23592557 PMC3662311

[R96] Osorio FG, Bárcena C, Soria-Valles C, Ramsay AJ, de Carlos F, Cobo J, Fueyo A, Freije JMP, López-Otín C, 2012. Nuclear lamina defects cause ATM-dependent NF-κB activation and link accelerated aging to a systemic inflammatory response. Genes Dev. 26, 2311–2324. 10.1101/gad.197954.112. 23019125 PMC3475803

[R97] Padhiar AA, Yang X, Zaidi SAA, Li Z, Liao J, Shu W, Chishti AA, He L, Alam G, Faqeer A, Ali I, Zhang S, Wang T, Liu T, Zhou M, Wang G, Zhou Y, Zhou G, 2024. MAM-STAT3-Driven mitochondrial ca ^+2^ upregulation contributes to immunosenescence in type a mandibuloacral dysplasia patients. Adv. Sci 10.1002/advs.202407398. PMC11791949 39661729

[R98] Paulin N, Viola JR, Maas SL, de Jong R, Fernandes-Alnemri T, Weber C, Drechsler M, Döring Y, Soehnlein O, 2018. Double-Strand DNA sensing Aim2 inflammasome regulates atherosclerotic plaque vulnerability. Circulation 138, 321–323. 10.1161/CIRCULATIONAHA.117.033098. 30012706

[R99] Pham PT, Fukuda D, Nishimoto S, Kim-Kaneyama J-R, Lei X-F, Takahashi Y, Sato T, Tanaka K, Suto K, Kawabata Y, Yamaguchi K, Yagi S, Kusunose K, Yamada H, Soeki T, Wakatsuki T, Shimada K, Kanematsu Y, Takagi Y, Shimabukuro M, Setou M, Barber GN, Sata M, 2021. STING, a cytosolic DNA sensor, plays a critical role in atherogenesis: a link between innate immunity and chronic inflammation caused by lifestyle-related diseases. Eur. Heart J 42, 4336–4348. 10.1093/eurheartj/ehab249. 34226923

[R100] Platanias LC, 2005. Mechanisms of type-I- and type-II-interferon-mediated signalling. Nat. Rev. Immunol 5, 375–386. 10.1038/nri1604. 15864272

[R101] Puttaraju M, Jackson M, Klein S, Shilo A, Bennett CF, Gordon L, Rigo F, Misteli T, 2021. Systematic screening identifies therapeutic antisense oligonucleotides for Hutchinson–Gilford progeria syndrome. Nat. Med 27, 526–535. 10.1038/s41591-021-01262-4. 33707772 PMC10167920

[R102] Reeh H, Rudolph N, Billing U, Christen H, Streif S, Bullinger E, Schliemann-Bullinger M, Findeisen R, Schaper F, Huber HJ, Dittrich A, 2019. Response to IL-6 trans- and IL-6 classic signalling is determined by the ratio of the IL-6 receptor α to gp130 expression: fusing experimental insights and dynamic modelling. Cell Commun. Signal 17, 46. 10.1186/s12964-019-0356-0. 31101051 PMC6525395

[R103] Ritchie C, Carozza JA, Li L, 2022. Biochemistry, cell biology, and pathophysiology of the innate immune cGAS–cGAMP–STING pathway. Annu Rev. Biochem 91, 599–628. 10.1146/annurev-biochem-040320-101629. 35287475

[R104] Rivera-Torres J, Acín-Perez R, Cabezas-Sánchez P, Osorio FG, Gonzalez-Gómez C, Megias D, Cámara C, López-Otín C, Enríquez JA, Luque-García JL, Andrés V, 2013. Identification of mitochondrial dysfunction in Hutchinson-Gilford progeria syndrome through use of stable isotope labeling with amino acids in cell culture. J. Proteom 91, 466–477. 10.1016/j.jprot.2013.08.008. 23969228

[R105] Rock KL, Latz E, Ontiveros F, Kono H, 2010. The sterile inflammatory response. Annu Rev. Immunol 28, 321–342. 10.1146/annurev-immunol-030409-101311. 20307211 PMC4315152

[R106] Ruppert M, Onodi ZS, Leszek P, Toth VE, Koncsos G, Merkely B, Ferdinandy P, Radovits T, Varga Z, 2020. AIM2-driven inflammasome activation in chronic heart failure. Eur. Heart J 41. 10.1093/ehjci/ehaa946.0847.

[R107] Saez A, Herrero-Fernandez B, Gomez-Bris R, Somovilla-Crespo B, Rius C, Gonzalez-Granado JM, 2020. Lamin A/C and the immune system: one intermediate filament, many faces. Int J. Mol. Sci 21, 6109. 10.3390/ijms21176109. 32854281 PMC7504305

[R108] Samson N, Ablasser A, 2022. The cGAS–STING pathway and cancer. Nat. Cancer 3, 1452–1463. 10.1038/s43018-022-00468-w. 36510011

[R109] Santiago-Fernández O, Osorio FG, Quesada V, Rodríguez F, Basso S, Maeso D, Rolas L, Barkaway A, Nourshargh S, Folgueras AR, Freije JMP, López-Otín C, 2019. Development of a CRISPR/Cas9-based therapy for Hutchinson–Gilford progeria syndrome. Nat. Med 25, 423–426. 10.1038/s41591-018-0338-6. 30778239 PMC6546610

[R110] Schlee M, Hartmann G, 2016. Discriminating self from non-self in nucleic acid sensing. Nat. Rev. Immunol 16, 566–580. 10.1038/nri.2016.78. PMC7097691 27455396

[R111] Scott LJ, 2017. Tocilizumab: a review in rheumatoid arthritis. Drugs 77, 1865–1879. 10.1007/s40265-017-0829-7. 29094311 PMC5736769

[R112] Sengupta D, Sengupta K, 2024. Lamin a K97E leads to NF-κB-mediated dysfunction of inflammatory responses in dilated cardiomyopathy. Biol. Cell 116. 10.1111/boc.202300094. 38404031

[R113] Shamseddine A, Patel SH, Chavez V, Moore ZR, Adnan M, Di Bona M, Li J, Dang CT, Ramanathan LV, Oeffinger KC, Liu JE, Steingart RM, Piersigilli A, Socci ND, Chan AT, Yu AF, Bakhoum SF, Schmitt AM, 2023. Innate immune signaling drives late cardiac toxicity following DNA-damaging cancer therapies. J. Exp. Med 220. 10.1084/jem.20220809. PMC9767651 36534085

[R114] Sharma M, de Alba E, 2023. Assembly mechanism of the inflammasome sensor AIM2 revealed by single molecule analysis. Nat. Commun 14, 7957. 10.1038/s41467-023-43691-4. PMC10693601 38042863

[R115] Shen H, Kreisel D, Goldstein DR, 2013. Processes of sterile inflammation. J. Immunol 191, 2857–2863. 10.4049/jimmunol.1301539. 24014880 PMC3787118

[R116] Shin J-Y, Worman HJ, 2022b. Molecular pathology of laminopathies. Annu. Rev. Pathol. Mech. Dis 17, 159–180. 10.1146/annurev-pathol-042220-034240. PMC8881990 34672689

[R117] Shin J-Y, Worman HJ, 2022a. Molecular pathology of laminopathies. Annu. Rev. Pathol. Mech. Dis 17, 159–180. 10.1146/annurev-pathol-042220-034240. PMC8881990 34672689

[R118] Soehnlein O, Tall AR, 2022. AIMing 2 treat atherosclerosis. Nat. Rev. Cardiol 19, 567–568. 10.1038/s41569-022-00755-0. 35882998 PMC9315844

[R119] Squarzoni S, Schena E, Sabatelli P, Mattioli E, Capanni C, Cenni V, D’Apice MR, Andrenacci D, Sarli G, Pellegrino V, Festa A, Baruffaldi F, Storci G, Bonafè M, Barboni C, Sanapo M, Zaghini A, Lattanzi G, 2021. Interleukin-6 neutralization ameliorates symptoms in prematurely aged mice. Aging Cell 20. 10.1111/acel.13285. PMC7811841 33393189

[R120] Srivastava RAK, Cornicelli JA, Markham B, Bisgaier CL, 2018. Gemcabene, a first-in-class lipid-lowering agent in late-stage development, down-regulates acute-phase C-reactive protein via C/EBP-δ-mediated transcriptional mechanism. Mol. Cell Biochem 449, 167–183. 10.1007/s11010-018-3353-5. 29644527 PMC6223808

[R121] Sun X, Duan J, Gong C, Feng Y, Hu J, Gu R, Xu B, 2022. Colchicine ameliorates dilated cardiomyopathy via SIRT2-Mediated suppression of NLRP3 inflammasome activation. J. Am. Heart Assoc 11. 10.1161/JAHA.122.025266. PMC9333380 35766262

[R122] Sun Y, Xu L, Li Y, Jia S, Wang G, Cen X, Xu Y, Cao Z, Wang J, Shen N, Hu L, Zhang J, Mao J, Xia H, Liu Z, Fu X, 2024. Mitophagy defect mediates the aging-associated hallmarks in Hutchinson–Gilford progeria syndrome. Aging Cell 23. 10.1111/acel.14143. PMC11296130 38482753

[R123] Suzuki M, Jeng LJB, Chefo S, Wang Y, Price D, Li X, Wang J, Li R-J, Ma L, Yang Y, Zhang X, Zheng N, Zhang K, Joseph DB, Shroff H, Doan J, Pacanowski M, Smpokou P, Donohue K, Joffe HV, 2023. FDA approval summary for lonafarnib (Zokinvy) for the treatment of Hutchinson-Gilford progeria syndrome and processing-deficient progeroid laminopathies. Genet. Med 25, 100335. 10.1016/j.gim.2022.11.003. 36507973

[R124] Takaki T, Millar R, Hiley CT, Boulton SJ, 2024. Micronuclei induced by radiation, replication stress, or chromosome segregation errors do not activate cGAS-STING. Mol. Cell 84, 2203–2213.e5. 10.1016/j.molcel.2024.04.017. 38749421

[R125] Unterholzner L, Keating SE, Baran M, Horan KA, Jensen SB, Sharma S, Sirois CM, Jin T, Latz E, Xiao TS, Fitzgerald KA, Paludan SR, Bowie AG, 2010. IFI16 is an innate immune sensor for intracellular DNA. Nat. Immunol 11, 997–1004. 10.1038/ni.1932. 20890285 PMC3142795

[R126] Vehns E, Arnold R, Djabali K, 2022. Impact of MnTBAP and baricitinib treatment on Hutchinson–Gilford progeria fibroblasts. Pharmaceuticals 15, 945. 10.3390/ph15080945. 36015093 PMC9415676

[R127] Wang X, Pan J, Liu H, Zhang M, Liu D, Lu L, Tian J, Liu M, Jin T, An F, 2019. AIM2 gene silencing attenuates diabetic cardiomyopathy in type 2 diabetic rat model. Life Sci. 221, 249–258. 10.1016/j.lfs.2019.02.035. 30790610

[R128] Weinreb JT, Ghazale N, Pradhan K, Gupta V, Potts KS, Tricomi B, Daniels NJ, Padgett RA, De Oliveira S, Verma A, Bowman TV, 2021. Excessive R-loops trigger an inflammatory cascade leading to increased HSPC production. e5 Dev. Cell 56, 627–640. 10.1016/j.devcel.2021.02.006. PMC8258699 33651979

[R129] Xian H, Watari K, Sanchez-Lopez E, Offenberger J, Onyuru J, Sampath H, Ying W, Hoffman HM, Shadel GS, Karin M, 2022. Oxidized DNA fragments exit mitochondria via mPTP- and VDAC-dependent channels to activate NLRP3 inflammasome and interferon signaling. e8 Immunity 55, 1370–1385. 10.1016/j.immuni.2022.06.007. 35835107 PMC9378606

[R130] Xiong L, Zhao K, Cao Y, Guo H-H, Pan J-X, Yang X, Ren X, Mei L, Xiong W-C, 2020. Linking skeletal muscle aging with osteoporosis by lamin A/C deficiency. PLoS Biol. 18, e3000731. 10.1371/journal.pbio.3000731. 32479501 PMC7310860

[R131] Xiong Y, Leng Y, Tian H, Deng X, Li Wenyuan, Li Wei, Xia Z, 2023. Decreased MFN2 activates the cGAS-STING pathway in diabetic myocardial ischaemia–reperfusion by triggering the release of mitochondrial DNA. Cell Commun. Signal 21, 192. 10.1186/s12964-023-01216-y. 37537600 PMC10398939

[R132] Xiong ZM, Choi JY, Wang K, Zhang H, Tariq Z, Wu D, Ko E, Ladana C, Sesaki H, Cao K, 2016. Methylene blue alleviates nuclear and mitochondrial abnormalities in progeria. Aging Cell 15, 279–290. 10.1111/acel.12434. PMC4783354 26663466

[R133] Xue C, Yao Q, Gu X, Shi Q, Yuan X, Chu Q, Bao Z, Lu J, Li L, 2023. Evolving cognition of the JAK-STAT signaling pathway: autoimmune disorders and cancer. Signal Transduct. Target Ther 8, 204. 10.1038/s41392-023-01468-7. 37208335 PMC10196327

[R134] Yan M, Li Y, Luo Q, Zeng W, Shao X, Li L, Wang Q, Wang D, Zhang Y, Diao H, Rong X, Bai Y, Guo J, 2022. Mitochondrial damage and activation of the cytosolic DNA sensor cGAS–STING pathway lead to cardiac pyroptosis and hypertrophy in diabetic cardiomyopathy mice. Cell Death Discov. 8, 258. 10.1038/s41420-022-01046-w. PMC9091247 35538059

[R135] Yang Y, Wang H, Kouadir M, Song H, Shi F, 2019. Recent advances in the mechanisms of NLRP3 inflammasome activation and its inhibitors. Cell Death Dis. 10, 128. 10.1038/s41419-019-1413-8. 30755589 PMC6372664

[R136] Yu Y, Liu Y, An W, Song J, Zhang Y, Zhao X, 2018. STING-mediated inflammation in kupffer cells contributes to progression of nonalcoholic steatohepatitis. J. Clin. Investig 129, 546–555. 10.1172/JCI121842. 30561388 PMC6355218

[R137] Zeng C, Duan F, Hu J, Luo B, Huang B, Lou X, Sun X, Li H, Zhang X, Yin S, Tan H, 2020. NLRP3 inflammasome-mediated pyroptosis contributes to the pathogenesis of non-ischemic dilated cardiomyopathy. Redox Biol. 34, 101523. 10.1016/j.redox.2020.101523. 32273259 PMC7327979

[R138] Zhang Y-G, Lu R, Wu S, Chatterjee I, Zhou D, Xia Y, Sun J, 2020. Vitamin d receptor protects against dysbiosis and tumorigenesis via the JAK/STAT pathway in intestine. Cell Mol. Gastroenterol. Hepatol 10, 729–746. 10.1016/j.jcmgh.2020.05.010. 32497792 PMC7498955

[R139] Zhang Z, Zhang C, 2025. Regulation of cGAS–STING signalling and its diversity of cellular outcomes. Nat. Rev. Immunol 10.1038/s41577-024-01112-7. 39774812

[R140] Zheng D, Liwinski T, Elinav E, 2020. Inflammasome activation and regulation: toward a better understanding of complex mechanisms. Cell Discov. 6, 36. 10.1038/s41421-020-0167-x. PMC7280307 32550001

[R141] Zhong Z, Liang S, Sanchez-Lopez E, He F, Shalapour S, Lin X, Wong J, Ding S, Seki E, Schnabl B, Hevener AL, Greenberg HB, Kisseleva T, Karin M, 2018. New mitochondrial DNA synthesis enables NLRP3 inflammasome activation. Nature 560, 198–203. 10.1038/s41586-018-0372-z. 30046112 PMC6329306

[R142] Zhou H, Wang X, Xu T, Gan D, Ma Z, Zhang H, Zhang J, Zeng Q, Xu D, 2024. PINK1-mediated mitophagy attenuates pathological cardiac hypertrophy by suppressing the mtDNA release-activated cGAS-STING pathway. Cardiovasc Res. 10.1093/cvr/cvae238. 39498806

